# Separation of U87 glioblastoma cell-derived small and medium extracellular vesicles using elasto-inertial flow focusing (a spiral channel)

**DOI:** 10.1038/s41598-022-10129-8

**Published:** 2022-04-12

**Authors:** Farhad Shiri, Haidong Feng, Kevin E. Petersen, Himanshu Sant, Gina T. Bardi, Luke A. Schroeder, Michael L. Merchant, Bruce K. Gale, Joshua L. Hood

**Affiliations:** 1 Department of Mechanical Engineering, University of Utah, Salt Lake City, UT 84112 USA; 2 Department of Pharmacology and Toxicology, School of Medicine, University of Louisville, Louisville, KY 40202 USA; 3 Brown Cancer Center, University of Louisville, Louisville, KY 40202 USA; 4 Hepatobiology and Toxicology COBRE, University of Louisville, Louisville, KY 40202 USA; 5 Division of Nephrology and Hypertension, Department of Medicine, University of Louisville, Louisville, KY 40202 USA

**Keywords:** Biomarkers, Biomedical engineering, Microfluidics, Mechanical engineering

## Abstract

Nanoscale and microscale cell-derived extracellular vesicle types and subtypes are of significant interest to researchers in biology and medicine. Extracellular vesicles (EVs) have diagnostic and therapeutic potential in terms of biomarker and nanomedicine applications. To enable such applications, EVs must be isolated from biological fluids or separated from other EV types. Developing methods to fractionate EVs is of great importance to EV researchers. Our goal was to begin to develop a device that would separate medium EVs (mEVs, traditionally termed microvesicles or shedding vesicles) and small EVs (sEVs, traditionally termed exosomes) by elasto-inertial effect. We sought to develop a miniaturized technology that works similar to and provides the benefits of differential ultracentrifugation but is more suitable for EV-based microfluidic applications. The aim of this study was to determine whether we could use elasto-inertial focusing to re-isolate and recover U87 mEVs and sEVs from a mixture of mEVs and sEVs isolated initially by one round of differential ultracentrifugation. The studied spiral channel device can continuously process 5 ml of sample fluid per hour. Using the channel, sEVs and mEVs were recovered and re-isolated from a mixture of U87 glioma cell-derived mEVs and sEVs pre-isolated by one round of differential ultracentrifugation. Following two passes through the spiral channel, approximately 55% of sEVs were recovered with 6% contamination by mEVs (the recovered sEVs contained 6% of the total mEVs). In contrast, recovery of U87 mEVs and sEVs re-isolated using a typical second centrifugation wash step was only 8% and 53%, respectively. The spiral channel also performed similar to differential ultracentrifugation in reisolating sEVs while significantly improving mEV reisolation from a mixture of U87 sEVs and mEVs. Ultimately this technology can also be coupled to other microfluidic EV isolation methods in series and/or parallel to improve isolation and minimize loss of EV subtypes.

## Introduction

Extracellular vesicles (EVs) play an important role in different biological functions. They function as intercellular messengers^1,2^. The RNAs and protein molecules in EVs serve as powerful mediators capable of modifying the behavior of cells, both locally and distally. Small EVs (sEVs), traditionally termed exosomes, are ~ 30 to 200 nm diameter, and formed intracellularly within multivesicular bodies (MVBs)^3^. MVBs then fuse with the inner leaflet of the plasma membrane, releasing exosomes or sEVs into the external cellular space^1,4,5^.

Oncosomes are cancer cell-derived shedding vesicles or microvesicles^3^. They are medium EVs (mEVs) with a size range of 100–1000 nm^3^. They are produced as a result of cell membrane zeiosis and fission. Research has shown that tumor progression can be accelerated by way of intercellular signaling that occurs between cancer cells and other cells in the tumor microenvironment. Both mEVs and sEVs participate in this communication process facilitating tumor progression^3,6,7^.

Several commercial techniques exist for collecting sEVs. Methods have been developed to isolate sEVs based on biophysical properties including size, solubility, and density. Among the separation methods, ultrafiltration is one common, fast, and inexpensive size-based technique^8,9^. Flow field-flow fractionation^10,11^ is another size-based separation technique used for sEV isolation. The major drawback associated with these techniques is their low recovery because of membrane adsorption of samples. In precipitationbased sEV isolation, sEVs are isolated from biological fluids by altering their dispersibility or solubility, essentially sequestering EVs from water^12,13^. Precipitation suffers from some disadvantages, including tedious pre- and post-processing steps. In addition, co-precipitation of other non-EV contaminating particles, such as proteins, lipoproteins or polymeric particles can occur during the precipitation process. Size separation techniques like size exclusion chromatography enable gentle separation of EVs based on size, but have difficulty distinguishing overlapping mEV and sEV subtypes of the same size and shape that differ in terms of density.

Beyond conventional EV isolation methods, microfluidics-based techniques have been proposed for the isolation of sEVs, such as porous silicon nanowire-on-micropillar separation^14^, acoustic microfluidic separation^15^, microfluidic coflow systems^16^, and elasto-inertial focusing using wavy microchannels^17^. One microfluidic approach with the potential to simultaneously process both sEVs and mEVs is inertial microfluidics. Inertial microfluidics is a passive and continuous microfluidics-based technique that has been widely used for the isolation of microparticles^18–27^. Inertial microfluidic approaches have several advantages, including simple device configurations, high throughput, and no need for an external field. Inertial approaches have gained significant attention since they were first demonstrated on the microscale in 2007^25^. Different microchannel geometries have been used to implement inertial focusing, including asymmetric serpentine^25–27^, symmetric serpentine^28–30^, pillar array^31,32^, straight^33–35^, and spiral configurations^18–21,36,37^. Tay et al.^38^ demonstrated the use of inertial focusing (a Spiral microchannel configuration) to isolate large microvesicles/microparticles (> 1 µm) from submicron microvesicles/microparticles (< 1 µm).

However, mEVs and sEVs can overlap in size, so separation via differential ultracentrifugation continues to be a viable option versus size alone techniques. Differential ultracentrifugation is recognized as the gold-standard technique for EV isolation^4,39,40^. EVs are isolated based on their density with sEVs typically isolated using a final ultracentrifugation step. Typically, a subsequent ultracentrifugation washing step is useful to minimize the presence of non-EV contaminants such as albumin in sEV preparations. Unfortunately, sEV recovery following the washing step can result in additional sEV damage and loss^40,41^.

Considering the afore mentioned EV isolation techniques and their limitations, our goal is to begin to develop a device that would separate mEVs and sEVs by elasto-inertial effect. The inertia of an object is directly proportional to its mass and density. The purpose of this work is not to replace differential ultracentrifugation for mEV and sEV isolation. We appreciate conventional ultracentrifuge EV isolation protocols and they are particularly effective at concentrating EVs from large sample volumes. Because we recognize the benefits of differential ultracentrifugation, our goal is to develop a more miniaturized technology that works like differential ultracentrifugation but is more suitable for microfluidic chip-based EV applications. We sought to couple the benefits of using differential ultracentrifugation to isolate mEVs and sEVs based on density while minimizing the loss associated with a second round of centrifugation to increase EV purity. We reasoned that the typical washing step could be replaced with gentler (less applied g force) inertial microfluidics processing. The aim of this study is to determine whether we can re-separate and recover U87 mEVs and sEVs pre-isolated by one round of differential ultracentrifugation using elasto-inertial focusing.

Our proposed spiral channel elasto-inertial focusing device is designed to differentially focus, as described below, mEVs and sEVs. The difference in EV focusing status results in mEV and sEV separation. The long-term objective of this work is to develop a technique for high-throughput isolation and collection of mEVs and sEVs that might be used independently or coupled to existing EV isolation technologies such as differential ultracentrifugation, buoyant density gradient separation, size exclusion chromatography, precipitation and other techniques to expand the tool set available to isolate and interrogate different EV types and subtypes.

### Elasto-inertial focusing theory

With a high flow rate, inertial force has influence on particle movement. Particles will have cross-streamline movement instead of moving along streamlines. In the microchannel with pressure-driven flow, the large velocity gradient leads to the generation of a shear lift force that pushes particles towards channel sidewalls. This phenomenon is defined as inertial focusing. This effect is balanced by the wake formed by the wall effect that pushes the particles away. These two opposing forces result in the lateral migration of particles until an equilibrium is reached, forming particle focusing streams a specific distance from the channel wall. In a circular channel cross-section, particle focusing positions form an annulus away from the wall^42^. In square channel cross-sections, particles occupy four focusing positions in the middle of each sidewall. In rectangular channels, the focus reduces to two positions located in the middle of the long sidewall.

The particle focusing positions can be affected by other forces such as a drag force caused by secondary flow. In microchannels with a curvature, such as occurs in spiral microchannels, two vortices perpendicular to the primary flow are produced, which are called the Dean flow. The Dean flow is caused by differential path lengths along the channel and inertial drift of the high velocity flows in the center of the channel towards the wall with the larger radius. These flows lead to counter-rotating vortices as the flow makes up for this inertial movement. The Dean vortices do not contribute to the inertial focusing process, but they lead to lateral displacement of the focused particles, moving them to new focusing positions^19,29,33^. In rectangular channels, particle focus positions typically migrate towards the channel inner sidewall as a result of the Dean drag force. The particles are pushed away from the center of the channel and closer to the wall across the thin dimension of the channel.

The inertial focusing phenomenon discussed above is observed in Newtonian flow. Additionally, when viscoelastic fluid is used in the microchannel, another phenomenon called viscoelastic focusing is also observed. In viscoelastic flow, an uneven shear stress distribution is observed in the channel cross-section, which results in particle migration towards the channel centerline. In the presence of Dean flow, particles focused near the centerline migrate toward the wall^43–45^.

Overall, the movement of particles in inertial focusing microchannels is influenced by a combination of inertial effects, the Dean drag force, and elastic effects, which are represented by the dimensionless numbers of Re, De, and Wi, respectively^43^. These dimensionless numbers are expressed as:

1 $$Re= \frac{U{\rho }_{f}{\text{D}}_{\text{h}}}{\eta }$$

2 $$\text{De = Re}{\left(\frac{{\text{D}}_{\text{h}}}{2R}\right)}^{0.5}$$

3 $$Wi= \lambda \dot{\gamma }=\frac{2\lambda U}{{\text{D}}_{\text{h}}}$$

where ${\rho }_{f}$ is the density of the fluid, $U$ is the average velocity, $\eta$ is the viscosity of the fluid, $R$ is the radius of curvature of the channel, $\lambda$ is the fluid relaxation time, $\dot{\gamma }$ is the fluid shear rate over the channel cross-section, and ${\text{D}}_{\text{h}}$ is the hydrodynamic diameter. For a rectangular channel cross-section, ${\text{D}}_{\text{h}}$ can be calculated using

4 $${\text{D}}_{\text{h}}= \frac{2wh}{(w+h)}$$

where *w* and *h* represent the width and height of the channel.

The magnitude of the nontrivial lateral lift force ($${F}_{L})$$ that is the composition of the wall-effect-induced lift force $$({F}_{LW})$$ and the shear-gradient lift force ($${F}_{LS)}$$. $${F}_{L}$$ can be defined as^46^:

5 $${F}_{L }= \frac{{f}_{L}{\rho }_{f}{U}^{2}{d}^{4}}{{h}^{2}}$$

where ${f}_{L}$ is the lift coefficient, and $d$ is the diameter of the particles.

The Dean drag force (${F}_{D}$), generated by two counter-rotating Dean vortices is given by ^46^:

6 $${F}_{D}=3\pi \eta d{U}_{D}\sim \frac{{\rho }_{f}{U}^{2}d{{\text{D}}_{\text{h}}}^{2}}{R}$$

where ${U}_{D}$ is the magnitude of the Dean flow.

The elastic force (${F}_{E}$) exerted on the particles is caused by the first normal stress difference N_1_ = σ_xx_ – σ_yy_, which can be expressed as^46^:

7 $${F}_{E}= {C}_{E}{d}^{3}\nabla {N}_{1}\sim {d}^{3}\left(\nabla {\tau }_{11}-\nabla {\tau }_{22}\right)\sim {d}^{3}\lambda {\left(\frac{U}{w}\right)}^{3}$$

where ${C}_{E}$ is the non-dimensional elastic lift coefficient, ${N}_{1}$ is the tension along the main flow direction, and ${\tau }_{11}$ and ${\tau }_{22}$ are the normal stresses in the flow and velocity gradient directions, respectively.

**Figure 1:**
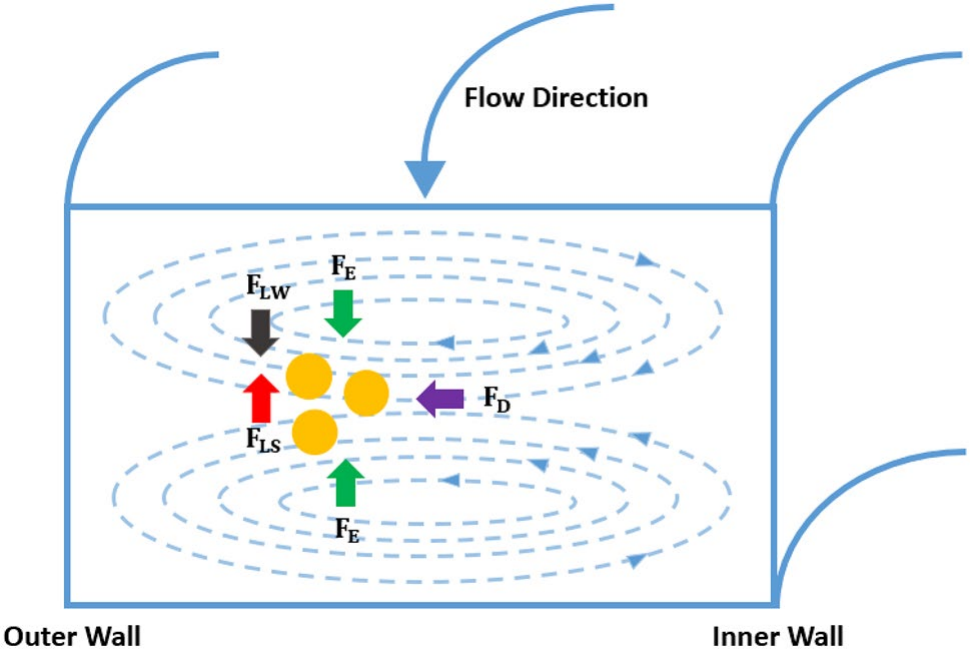
Mechanism of elasto-inertial focusing of particles in the spiral channel. $${F}_{LW}$$ and $${F}_{LS}$$ represent wall-effect-induced lift force and shear-gradient lift force, respectively. These two forces are the components of the nontrivial lateral lift force ($${F}_{L})$$. $${F}_{E}$$ and $${F}_{D}$$ represent elastic force and Dean drag force, respectively.

In viscoelastic flow, when Re is high, particle movement is affected by both the viscoelastic focusing and inertial focusing effects. The balance between elastic force and inertial force will determine the particle focusing position. If the elastic force dominates over the Dean drag force and the inertial force, the particles tend to focus at the centerline of the channel. As the elastic force weakens, the particles tend to be equilibrated at the outer region of the channel^43^. Based on the balance between the two types of forces and the resulting particle focusing locations, the separation of particles with different sizes can be realized. The mechanism of elasto-inertial flow focusing of particles is illustrated in Fig. 1.

## Experimental section

### Materials

28 nm (blue fluorescent, part number F8781) and 1000 nm (yellow-green fluorescent, part number F8823) diameter carboxylate-modified polystyrene (Thermo Fisher Scientific Inc, UT, USA) were used as test particles given their similarity in size to sEVs and mEVs, respectively. sEVs and mEVs were purified from the supernatants of a cultured U87 human glioma cancer cell line using standard ultracentrifugation techniques at the University of Louisville. Briefly, mEVs/microparticles were isolated post 10,000 *g* and sEVs isolated post 110,000 *g* according to established methods^2^. After the isolation process, fluorescent lipophilic cationic carbocyanine dyes were used to dye biological particles. 1,1'-dioctadecyl-3,3,3',3'-tetramethylindocarbocyanine perchlorate (DiI) and 3,3'-dioctadecyloxacarbocyanine perchlorate (DiO) were used to dye U87 sEVs and mEVs, respectively. Samples were frozen, shipped overnight, and stored at −80 °C until they were used. The received sEVs and mEVs were in 1× PBS buffer and had protein mass concentrations of 0.73 µg/ml and 0.12 µg/ml, respectively, as determined using a PierceTM BCA Protein Assay Kit (Thermo ScientificTM cat#23227, Waltham, MA, USA). A 1:10 dilution of received samples was made by adding one part stock to nine parts diluent (1× PBS buffer) for both sEVs and mEVs. Detailed information regarding the preparation of EVs is included in the Supplementary Information. Polyethylene oxide (PEO) dissolved in 1× PBS was used as the viscoelastic reagent in the spiral channel focusing tests. The PEO solution was prepared by mixing PEO powder that has a molecular weight of 2 × 10^6^ g/mol (Sigma-Aldrich, St. Louis, MO) with PBS. PEO concentrations ranging from 0.01 to 0.03% were used in the experiments. The polymer solution overlap concentration c* is estimated by measuring the intrinsic viscosity utilizing a capillary viscometer, c* = 1.1 mg/ml^47^. The PEO solutions used in these experiments were considered dilute as the PEO concentration was less than c*. The PEO solution relaxation time was estimated by Zimm theory^48^. The properties of the PEO solution are listed in Table 1.

**Table 1:** Properties of PEO solution.

Solution	c/c* (–)	Viscosity (cp)	Relaxation time λ (ms)
PBS	0	1.045	–
0.01% PEO	0.091	1.183	2.85
0.03% PEO	0.275	1.404	5.68

### Instrumentation

**Figure 2:**
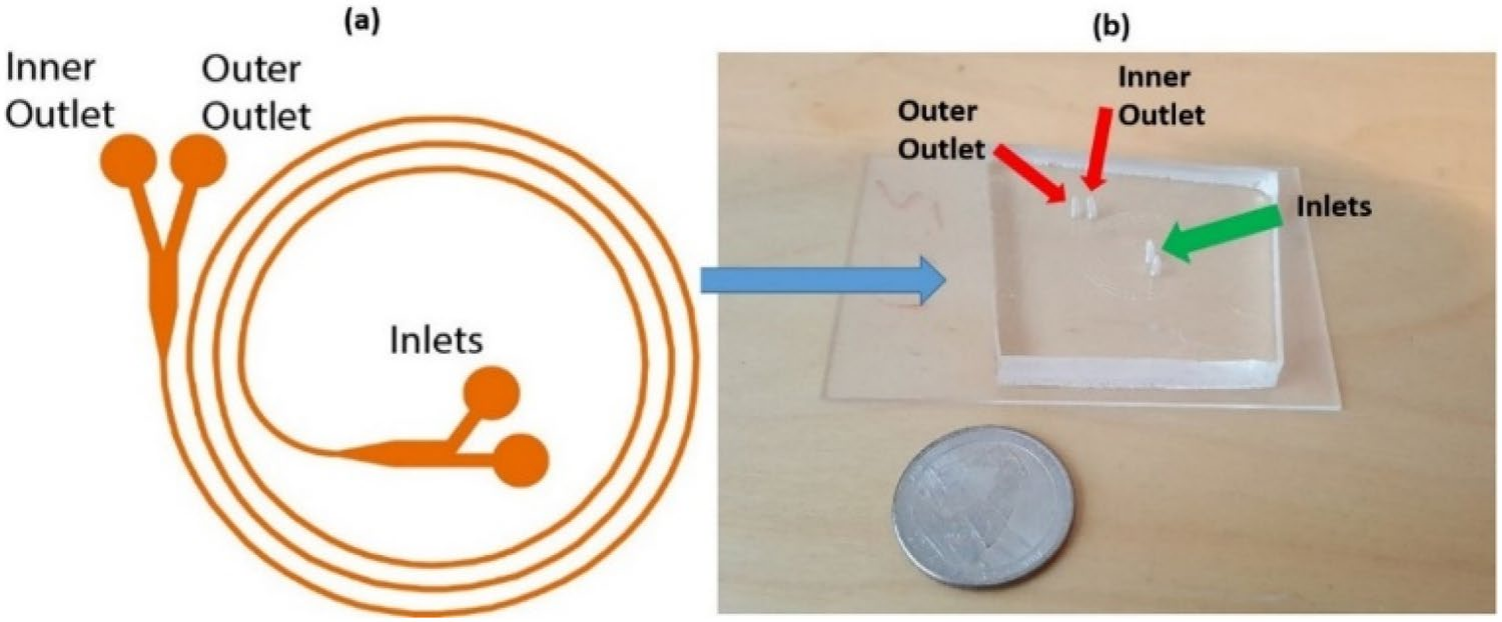
Spiral channel used for separation of sEVs and mEVs (**a**) the design (**b**) the fabricated device.

The spiral channel used in this work had a 100 µm width and 25 µm height with 7 to 9 mm radius and 3 loops. A picture of the design and the fabricated device is shown in Fig. 2. A Vanguard fluorescence microscope model 1486FL coupled with an AmScope microscope camera was used to take the images of the fluorescent particles through the inertial focusing tests. Two Dionex RF-2000 fluorescence detectors (Dionex Softron GmbH, Germany) were used for the detection of exiting particles at the outlet ports of the spiral channel. One of the detectors was set at the excitation wavelength of 365 nm and the emission wavelength of 415 nm for the detection of blue-dyed 28 nm particles. The other fluorescence detector was set at the excitation wavelength of 505 nm and the emission wavelength of 550 nm for the detection of yellow green dyed 1000 nm particles. For the series of tests done on the sEVs and mEVs, one of the detectors was set at the excitation wavelength of 549 nm and the emission wavelength of 570 nm in order to detect DiI dyed sEVs and the other detector was set at the excitation and emission wavelengths of 475 and 505 nm, respectively for detection of DiO dyed mEVs. The sensitivity and gain factors of the fluorescence detector used for the sEVs were set to 1 and 1, respectively. For the detector used for the mEVs, the sensitivity and gain factors were 1 and 2, respectively. A KD Scientific syringe pump model 210 (KD Scientific Inc., MA, USA) was used for the continuous injection of the sample into the device. The dynamic light scattering (DLS) size measurements of EVs were conducted using a NanoBrook 90Plus (Brookhaven Instruments Corporation, Holtsville, NY, USA).

## Methods

### Dynamic light scattering (DLS) analysis of extracellular vesicles

Dynamic light scattering (DLS) size measurements of EVs were obtained using a NanoBrook 90Plus (Brookhaven Instruments Corporation, Holtsville, NY, USA) incorporating a 40 mW, 640 nm, temperature-controlled red semiconductor laser, according to established methods^49–51^. For each measurement, 100 µL of the sample was analyzed at room temperature (25 °C) following dilution in 2 ml of 0.2 micron filtered 1× PBS within a disposable 4.5 ml capacity Brookhaven Instruments square plastic cuvette (BI-SCP) designed for aqueous samples. The hydrodynamic diameter of the EVs was determined at a 90° scattering angle based on the Stokes–Einstein equation and the Smoluchowski model for aqueous samples.

### Nanoparticle tracking analysis (NTA) of extracellular vesicles

Independent freshly isolated batches were isolated by differential ultracentrifugation in 1× PBS as described in the EV culture and isolation methods section. Freshly isolated EVs were stored in 0.5 ml of 1× PBS at −20 °C for 4 days prior to NTA. NTA was performed using an automated Particle Metrix ZetaView® BASIC NTA—Nanoparticle Tracking Video Microscope PMX-120. Analysis parameters included a maximum area set to 1000, factory optimized for EVs, minimum area set to 10 and minimum brightness of 30. For NTA, isolated sEVs and mEVs were diluted 1:1000 in Invitrogen ultrapure distilled water. Reisolated sEVs and mEVs were diluted 1:1000 and 1:100, respectively, given substantial loss of mEVs following re-isolation. EVs were traced using a laser wavelength of 520 nm in scatter mode with 11 position profiling.

### Transmission electron microscopy (TEM) of extracellular vesicles

Copper carbon formvar grids were glow discharged immediately prior to loading with mEVs (150 μg EV protein/0.331 ml in 50 mM trehalose in 1× PBS) or sEVs (150 μg EV protein/0.131 ml in 50 mM trehalose in 1× PBS) as determined using a PierceTM BCA Protein Assay Kit (Thermo ScientificTM cat#23227, Waltham, MA, USA). EVs were processed undiluted. Grids were floated on 10 μL EV sample drops for 15 min, washed two times with water by floating on the drop of water for 30 s, and negatively stained with 2% uranyl acetate by floating on the drop of stain for 30 s. The grid was blot dried with Whatman paper and imaged with a Jeol 1230 electron microscope followed by size distribution analysis of the imaged EVs.

### Fabrication of spiral microchannel

To fabricate polydimethylsiloxane (PDMS) spiral channel devices, the following procedure was used. First, a stamp or mold of the spiral channel was fabricated using standard photolithographical methods with SU-8 on a silicon wafer^52,53^. The Sylgard 184 PDMS curing agent and the base agent were mixed in a ratio of 1:10. 4 ml of curing agent was mixed with 40 ml of the base agent, and the mixture was placed in a vacuum chamber for 40 min in order to degas the mixture. The degassed mixture was then poured gently on the mold, and then the mold and liquid PDMS were placed on a heater at 150 °C for 20 min in order to cure the PDMS. Next, the cured PDMS was gently peeled off from the mold. A corona discharge treater model LM4243-05 (Enecron Industries Corp., WI, USA) was used to enable bonding between the PDMS and a glass sheet. The plasma was applied on the PDMS surface to be bonded and the glass sheet for 3 min before placing the two surfaces in contact. The final device produced after the bonding process is shown in Fig. 2.

### Separation of particles

The ability of the spiral channel to continuously separate sEVs from mEVs was investigated. A series of experiments were designed and performed to show the flow focusing of mEVs (as sEVs are too small to focus under the experimental conditions). The tests were designed to collect each particle type at the end of the channel, with the purity of the sEVs and mEVs varied by controlling the relative fluid outlet rates at the exit. This work utilizes the ability of the device to selectively focus the larger particles, thereby effecting a good separation between polystyrene test particles of different sizes. The sample sEVs and mEVs were sourced from U87 glioblastoma cell culture. U87 cells are an ideal model for developing sEV and mEV fractionation instruments since the cells readily produce abundant amounts of both sEVs and mEVs^54^. Since the biological sample source is limited, the conceptual demonstration was performed using polystyrene particles with sizes similar to the lower end of the size range reported for sEVs and the upper end of the size range reported for mEVs. Specifically, 28 nm (blue fluorescent) and 1000 nm (yellow-green fluorescent) diameter carboxylate-modified polystyrene particles were used. The polystyrene particles used for the optimization of the experimental conditions approximated the lower and upper size ranges reported for sEVs and mEVs, respectively. Theoretically, the obtained focusing conditions for the selected polystyrene particles should also work for the actual biological particles reported within the 28–1000 nm size range. In all experiments, the samples were injected continuously into both inlet ports and the flow rates of both inlet ports were equal. In the first test (denoted as B1), the focusing of 1000 nm yellow-green polystyrene particles was investigated. For this test, the sample was injected with a total flow rate of 0.08 ml/min. Viscoelastic polyethylene oxide (PEO) was mixed at a concentration of 0.01% by volume with the sample prior to injection. A similar test (denoted as B2) was done using the same sample and the same flow rate as test B1, but instead using a concentration of 0.03% PEO. The process of optimization of the flow and viscoelastic parameters for focusing of 1000 nm microbeads are included in the Supplementary Information.

For all focusing tests, particle focusing quality was evaluated by analyzing the images obtained from the fluorescent camera. The full width at half maximum (FWHM) of the focusing peak was calculated using MATLAB for each image.

After the tests on the 1000 nm polystyrene particles, experiment B3 consisted of a mixture of blue-dyed 28 nm and yellow-green 1000 nm particles injected using the same conditions as test B2. After running tests on polystyrene particles, the best condition for the separation of 28 nm and 1000 nm particles was selected and was applied to the biological samples (sEVs and mEVs). Test B4 used the B3 test condition with a mixture of sEVs and mEVs. A higher flow rate (total flow rate of 0.18 ml/min) was also applied and denoted test B5. The flow rate of 0.18 ml/min was the highest possible flow rate that the PDMS-Glass bonding of the fabricated spiral channel was able to withstand. While running test B5, the samples exiting from the inner and outer outlets were collected. After a sample run, collected samples were re-injected (recycling test B5R). For tests using fluorescence detectors, the percentage of exiting particles associated with each type of particle was calculated considering the obtained signal from each detector. The parameters used for the separation of particles are reported in Table 2.

**Table 2:** Table of tests.

Test number	Experiment test summary	Total channel flow rate (mL/min)	Channel exit splitting ratio (inner:outer)	Results shown in figure
B1	1000 nm PS with 0.01% PEO	0.08	50:50	4
B2	1000 nm PS with 0.03% PEO	0.08	50:50	5
B3	1000 nm PS with 28 nm PS with 0.03% PEO	0.08	50:50	6
B4	sEVs with mEVs with 0.03% PEO	0.08	50:50	7
B5	sEVs with mEVs with 0.03% PEO	0.18	50:50	8
B5R	sEV and mEV Outputs of B5 recycled as inputs to B5R with 0.03% PEO	0.18	50:50	9

## Results and discussion

### Characterization of EVs using DLS, NTA and TEM

**Figure 3:**
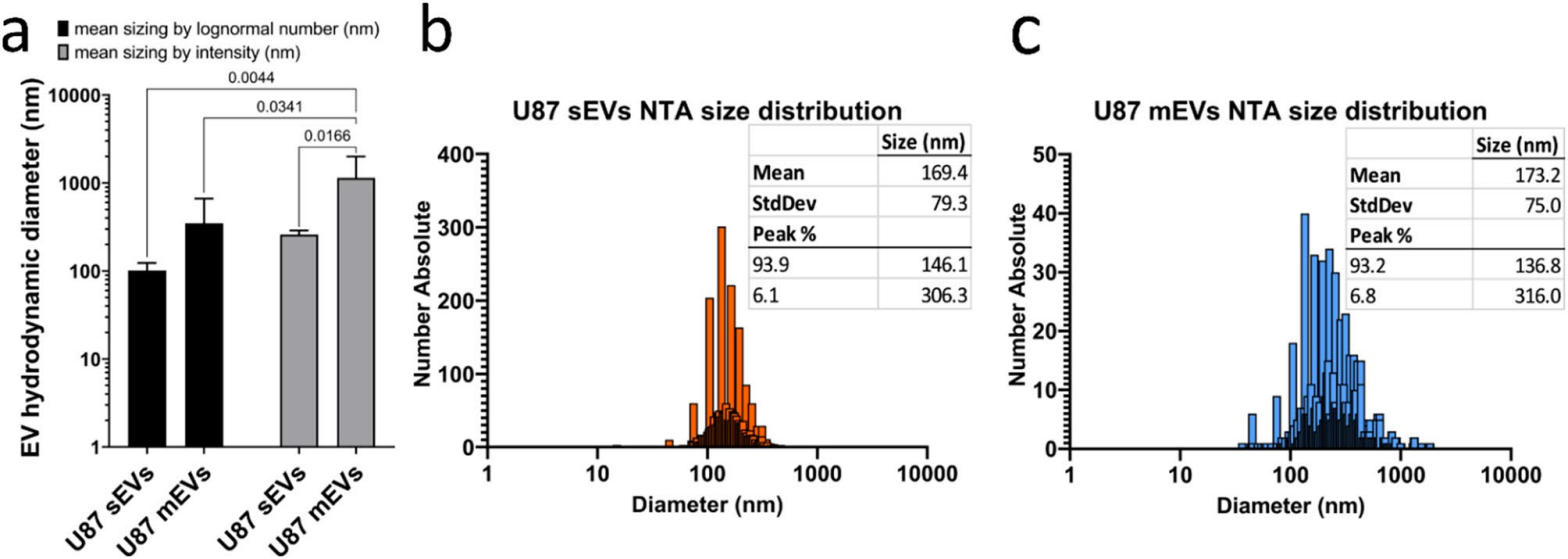
Characterization of U87 EV size by DLS and NTA. **(a)** EVs sized by dynamic light scattering using two different sizing modalities. Lognormal number detects the more numerous, smaller trending subpopulation of EVs in an EV pool, whereas intensity detects the larger sized subpopulation within an EV pool, error bars = SD (n = 6). Using 2-way ANOVA with p-values < 0.05 considered to be statistically significant, EV sizes were compared by type (sEV versus mEV, p = 0.007) and by DLS sizing modality (lognormal number versus intensity, p = 0.02). Tukey's Honest Significant Difference test was used post-hoc for multiple intra- and intergroup comparisons and p values < 0.05 are listed above interconnecting drop-down lines connecting bar plots. **(b)** sEV and **(c)** mEV size distributions determined by ZetaView nanoparticle tracking analysis including descriptive statistics tables.

The size of U87 sEVs and mEVs was measured by DLS using two different modes, including lognormal mean sizing by intensity and lognormal mean sizing by number (Fig. 3a). Lognormal mean sizing by intensity was operated to determine the upper size limit of the EVs or aggregates existing in a population, considering the fact that larger EVs scatter light more than smaller EVs. Additionally, lognormal mean sizing by number was used to obtain the lower size limit of individual EVs in a population as the number distribution highlights the most numerous, smaller size trending, particles in the distribution. Statistical analysis by 2-way ANOVA indicated significant differences (p < 0.05) in EV sizing when comparing EV types (sEV versus mEV) and comparing DLS sizing modalities (lognormal number versus intensity). Post-hoc statistical testing using Tukey's Honest Significant Difference test further demonstrated significant differences (p < 0.05) in EV size between U87 sEVs sized by lognormal number and U87 mEVs sized by intensity, U87 mEVs sized by lognormal number and U87 mEVs sized by intensity, and between U87 sEVs and mEVs sized by intensity. No significant difference was determined for U87 mEVs and sEVs sized by lognormal number. Taken together, the statistics demonstrate significant overlap in the sizes of the smaller, more numerous subpopulations within the separate U87 mEV and sEV pools, and a significant difference in size between the larger EV subpopulations found within the separate U87 mEV and sEV pools.

The measured U87 sEVs using lognormal mean sizing by number was consistent with the size range that is reported for sEVs in the literature (< 200 nm)^3,55^. The lognormal mean sizing by intensity measurements indicate that some of the sEVs were larger than 200 nm. This is possibly a result of the existence of larger sEVs in the sample, or it could be derived from the formation of sEV aggregates during the isolation process of EVs using ultracentrifugation at ~ 110,000*g*.

Measurement of the size of U87 mEVs using lognormal sizing by intensity revealed that some of the mEVs are larger than the reported upper 1000 nm size limit of mEVs (Fig. 3a)^3,55^. This could arise from the formation of aggregates during the isolation process of mEVs. As evidenced by lognormal number sizing, some of the mEVs are smaller than the reported lower size limit of mEVs (100 nm)^3,55^. This result could be caused by partial co-isolation of sEVs within the mEVs population during the ultracentrifugation process.

To further assess differences in U87 sEV and mEV size, EV size distributions were determined by nanoparticle tracking analysis (NTA) (Fig. 3b,c). U87 sEV and mEV size distributions of 169.4 ± 79.3 nm (Fig. 3b) and 173.2 ± 75.0 nm (Fig. 3c) respectively, were found to be consistent with those determined by DLS (Fig. 3a). NTA peak analysis revealed that sEVs contained predominantly smaller 146.1 nm sEVs at 93.9 peak % and larger 306.3 nm sEVs at 6.1 peak %. Similarly, U87 mEVs contained predominantly smaller 136.8 nm sEVs at 93.2 peak % and larger 316.0 nm sEVs at 6.8 peak %. NTA peak analysis for mEVs and sEVs was consistent with the detection of smaller EV subpopulations within the general sEV or mEV populations using mean sizing by lognormal number and larger EV subpopulations within the general sEV or mEV populations using mean sizing by intensity measurements determined by DLS (Fig. 3a).

**Figure 4:**
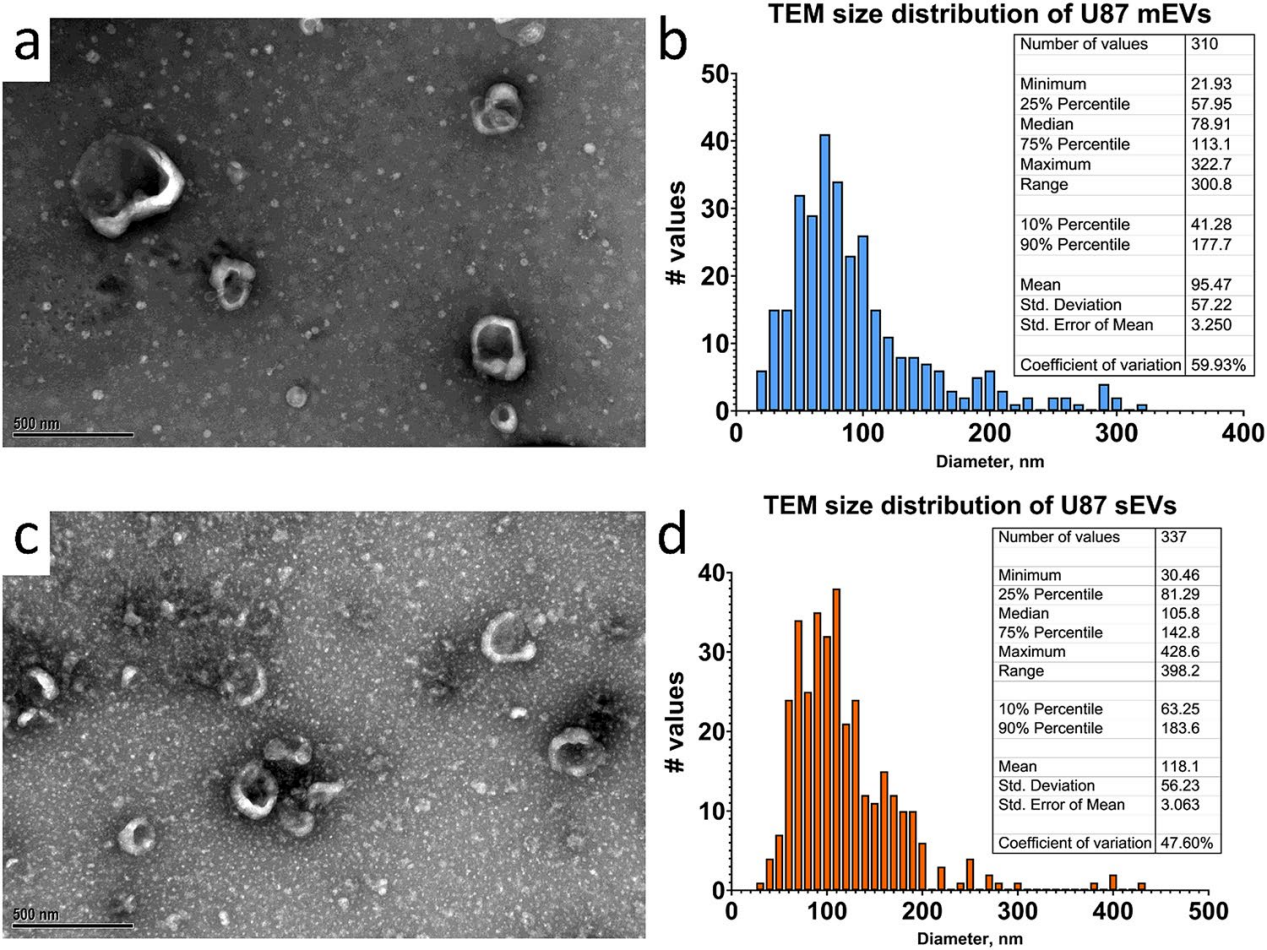
Characterization of U87 EV size by TEM. **(a)** Representative TEM of mEVs and **(b)** corresponding size distribution, **(c)** representative TEM of sEVs and **(d)** corresponding size distribution including descriptive statistics tables. The images of EV samples isolated in the laboratory of J.L. Hood were obtained through contract services with Alpha Nano Tech LLC (Morrisville, NC, USA).

Given the overlap in the size distributions of U87 mEVs and sEVs determined by DLS and NTA, TEM was used to directly visualize EVs (Fig. 4). TEM of U87 mEVs revealed a mixture of smaller and larger mEVs ranging in size from 21.93 to 322.7 nm (Fig. 4b). Although, the occasional mEV approaching 500 nm in size can also be observed (Fig. 4a). TEM of U87 sEV also revealed a mixture of smaller and large sEVs ranging in size from 30.46 to 398.2 nm (Fig. 4d). In contrast to the U87 mEVs, aggregates of sEVs are more frequently observed (Fig. 4c). Aggregates derived from ultracentrifugation processing may account for the larger sEV sizes calculated by TEM size distribution (Fig. 4d). TEM and NTA size distributions both showed a wider peak base for mEVs versus sEVs, with mEVs > 200 nm in size more apparent (Figs. 3c, 4b). However, the difference in mean mEV and sEV size determined by NTA versus TEM was approximately 51 and 78 nm, respectively. This difference is consistent with NTA measuring hydrodynamic EV diameter whereas TEM allows for better direct visualization of the actual EV diameter. EV hydrodynamic diameter is larger than actual diameter. However, dehydration of EVs during TEM sample processing can result in a smaller than actual EV diameter further increasing the observable difference in size between NTA and TEM measurements.

### Detection of EV relevant markers in U87 cells, mEVs and sEVs using LC–MS

**Figure 5:**
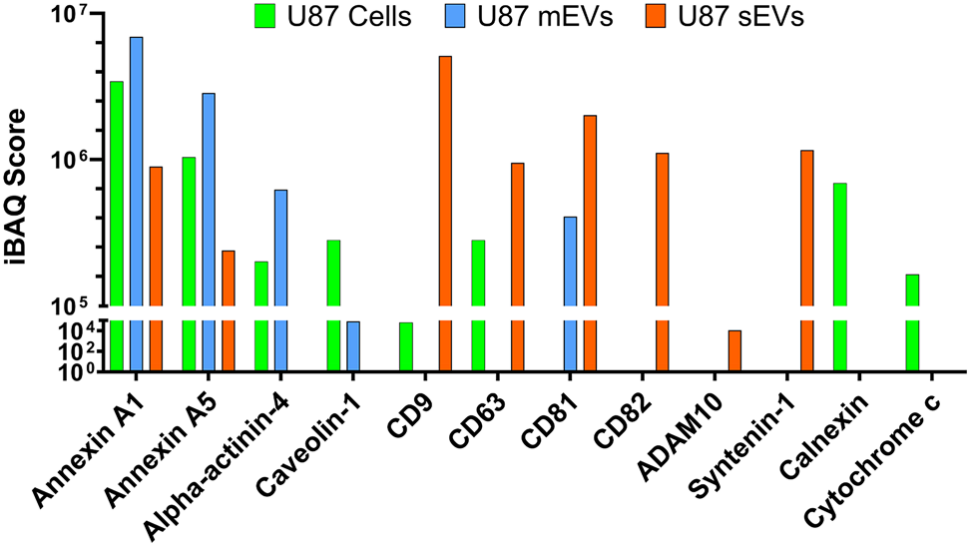
Identification of EV relevant markers in U87 cells and EVs by 1D-LCMS. The relative quantification is by intensity based absolute quantification (iBAQ). The absence of a bar indicates an iBAQ score of zero. A zero score indicates that the particular peptide was not observed in the sample or the software did not have the ability to quantify it from the local baseline signal.

To better characterize the U87 mEVs and sEVs in our study, we next sought to determine whether they contained reported microvesicle (shedding vesicle) and exosome markers respectively^56–59^. U87 EV source cells, mEVs and sEVs were analyzed by liquid chromatography mass spectrometry (LC–MS) (Fig. 5). LC–MS data analysis using Proteome Discoverer (ThermoFisher) determined that mEVs were enriched in shedding vesicle markers including Annexin A1, Annexin A5, alpha-Actinin-4, and Caveolin-1. In contrast, U87 sEVs were enriched in exosome markers including CD9, CD63, CD81, CD82, ADAM10 and Syntenin-1. The endoplasmic reticulum marker Calnexin and mitochondrial marker Cytochrome c were detected in U87 cells but not U87 mEVs or sEVs as expected.

### Determining U87 mEV and sEV recovery following re-isolation by centrifugation

**Figure 6:**
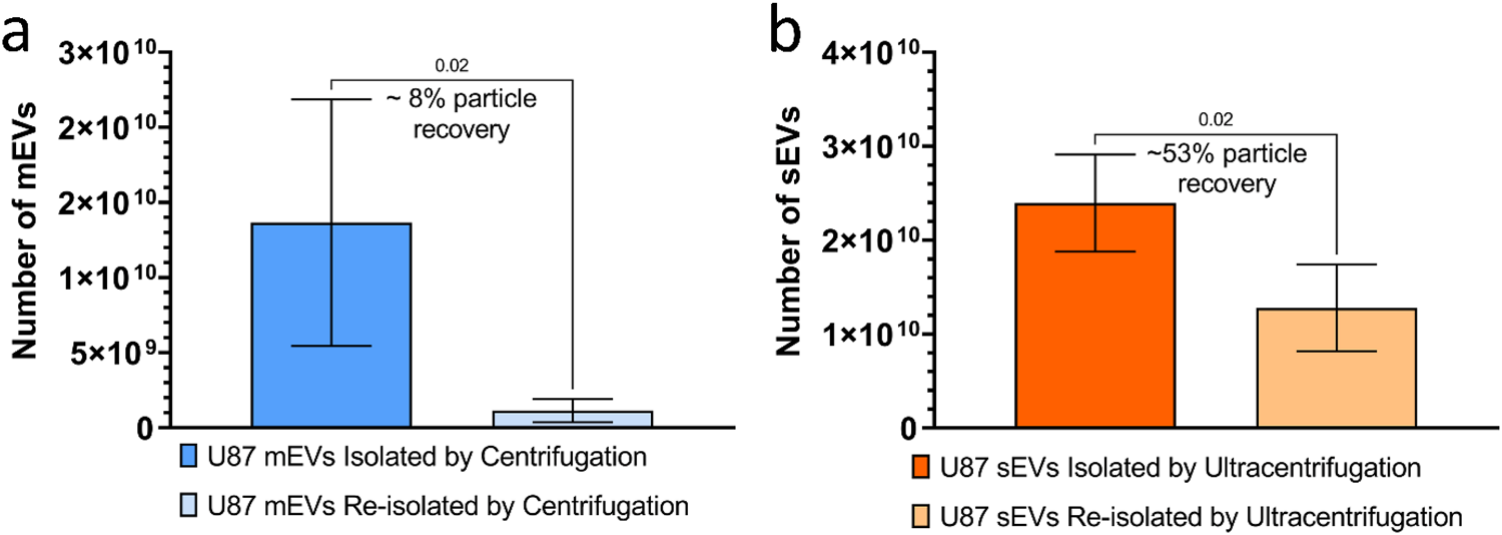
NTA quantification of EVs isolated and re-isolated by centrifugation. **(a)** U87 mEVs in 1× PBS were isolated by differential ultracentrifugation at 10,000×*g* and mEV particle yield calculated based on particle/ml concentrations determined by ZetaView NTA. Following quantification of particle yield, known quantities of mEVs were re-isolated by differential ultracentrifugation at 10,000×*g* and % particle recovery determined. **(b)** U87 sEVs in 1× PBS were isolated by differential ultracentrifugation at 110,000×*g* and sEV particle yield calculated based on particle/ml concentrations determined by ZetaView NTA. Following quantification of particle yield, known quantities of sEVs were re-isolated by differential ultracentrifugation at 110,000×*g* and % particle recovery determined. Error bars = SD for n = 4 independent EV isolations., p values, determined by Student’s t-test are listed above drop-down lines. p values < 0.05 were considered statistically significant.

Having extensively characterized the U87 mEVs and sEVs under study, we next sought to determine how many mEVs or sEVs could be recovered following re-isolation of mEVs using centrifugation or sEVs by ultracentrifugation. A re-isolation or washing step is commonly used to remove excess protein contaminants such as albumin from EV preparations. However, previous reports demonstrate repeat rounds of ultracentrifugation can result in sEV loss^40^. Using NTA to determine EV concentrations, we observed a significant loss of mEVs and sEVs following re-isolation (Fig. 6). An 8% and 53% particle recovery were determined for U87 mEVs and sEVs, respectively.

Given this low recovery combined with our data that U87 mEVs and sEVs overlap in size (Fig. 3), we next sought to isolate U87 mEVs and sEVs based on differences in their inertia. The inertia of an object is directly proportional to its mass and density. We reasoned that we might be able to mimic the ability of differential ultracentrifugation to effectively separate similar sized U87 mEVs and sEVs, based on differences in their density, using differences in U87 mEV and sEV inertia instead. To achieve this, mEVs and sEVs, isolated by differential ultracentrifugation, were labeled with fluorescent green (DiO-mEVs) and fluorescent red (DiI-sEVs), respectively. For purposes of comparison, we assigned a separation efficiency value to differential ultracentrifugation of 100%. So, we considered the two differentially labeled mEVs and sEVs to be 100% separated following one round of differential ultracentrifugation. We then pooled the two populations back together and proceeded to re-separate them using inertial focusing with a spiral channel to determine how closely we could achieve 100% re-separation.

### Separation of particles

**Figure 7:**
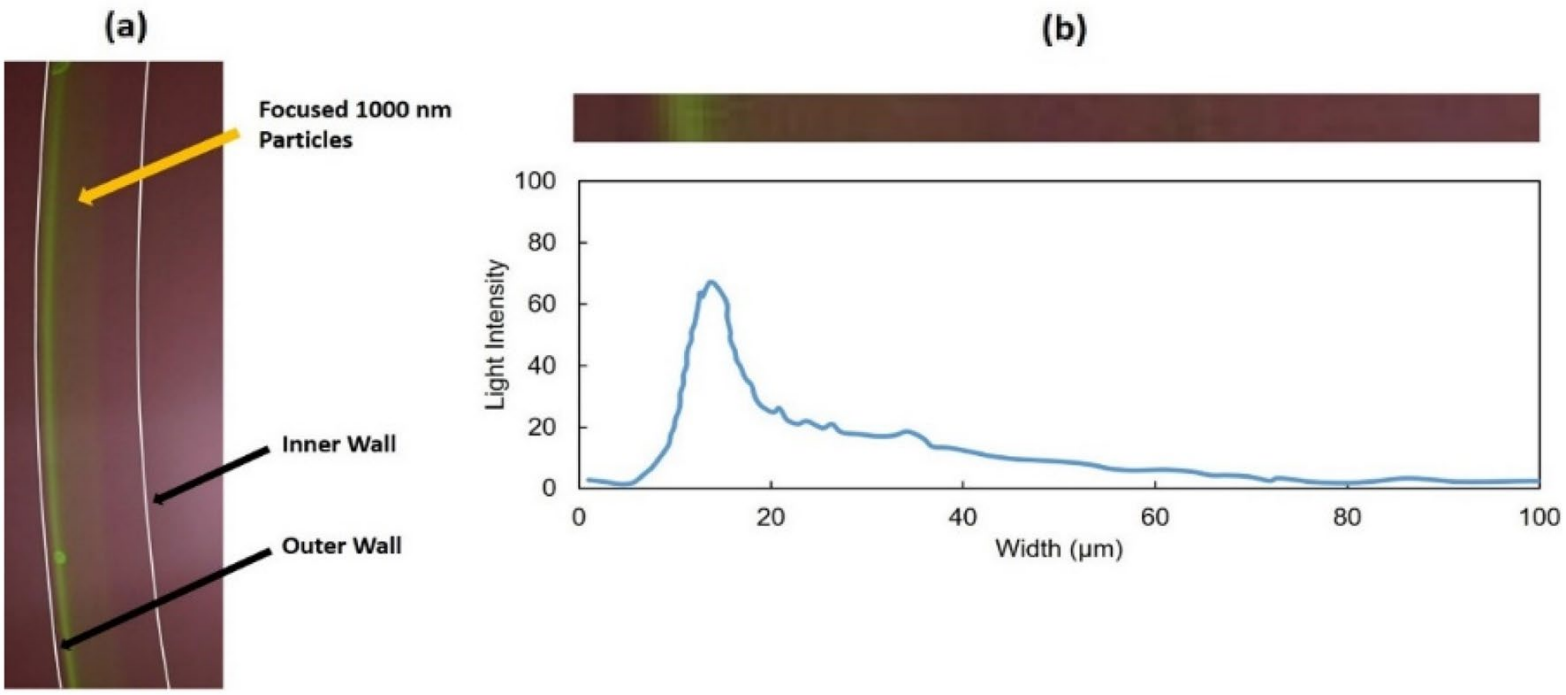
Results of 1000 nm polystyrene particles location imaging test (test B1). **(a)** Image taken by fluorescent microscope (the walls of the channel are marked by the white lines), **(b)** the light intensity across the width of the channel based on the image above the graph.

**Figure 8:**
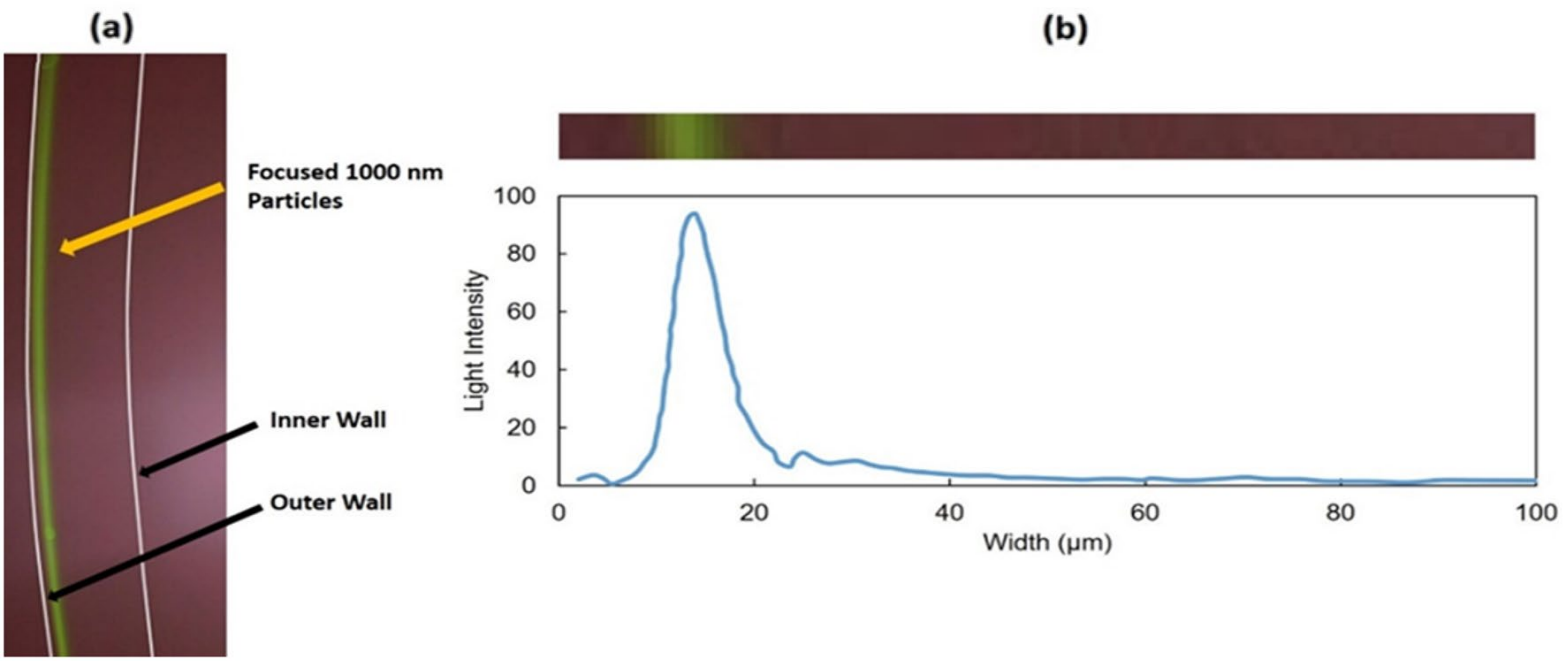
Results of 1000 nm polystyrene particles location imaging test (test B2). **(a)** Image taken by fluorescent microscope (the walls of the channel are marked by the white lines), **(b)** the light intensity across the width of the channel based on the image above the graph.

The results of test B1 with 1000 nm PS with 0.01% PEO are shown in Fig. 7. The image is taken near the outlet of the microchannel. The results indicate that a partially focused stream of 1000 nm particles appeared in the outer half of the channel. The image taken by a fluorescent microscope was analyzed, and the fluorescence distribution across the area was calculated (Fig. 7b). From the data in Fig. 7b, the calculated full width at half maximum (FWHM) for the focusing peak in test B1 was 7.1 µm. FWHM represents particle focusing quality. The lower FWHM, the better the focusing. Based on obtained light intensity distribution across the width of the channel, 82% of particles were focused within the half-width of the channel. The results of test B2 with 1000 nm PS with 0.03% PEO are shown in Fig. 8. The findings indicate that adding a higher concentration PEO improves the focusing of 1000 nm particles. Based on the image taken by the fluorescent microscope, an almost fully focused stream (note the elimination of most of the background) of 1000 nm particles appeared in the outer half of the channel. The calculated FWHM for this test was 5.9 µm. The lower FWHM compared to the previous test confirmed that improved focusing is achieved with a higher concentration of PEO. Based on the particle light intensity distribution observed across the width of the channel, 94% of particles were focused within the half-width of the channel.

**Figure 9:**
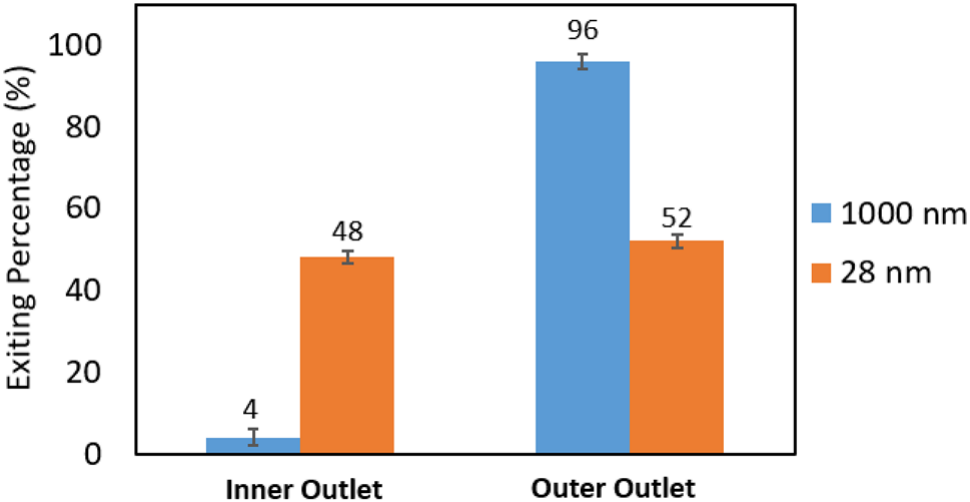
Results of an inertial focusing test on a mixture of 28 nm and 1000 nm polystyrene particles (test B3). Exiting percentage of particles from the inner outlet (left) and the outer outlet (right). n = 3, error bars = standard deviation (SD).

The results of test B3 evaluating 1000 nm PS, 28 nm PS, and 0.03% PEO are shown in Fig. 9. As expected, for the mixture of 28 nm and 1000 nm particles, the large particles were focused well in the outer half of the channel, and consequently, 96% of large particles exit the outer outlet while a small portion of those particles (4%) exit the inner outlet. As expected, no focusing was demonstrated for the 28 nm particles. The 28 nm particles were distributed across the width of the channel equally resulting in nearly the same exiting percentage through the inner and outer outlets. Based on the PS particle focusing results, mEVs, like 1000 nm PS, also will be driven to the outer outlet, and sEVs, similar to 28 nm PS, will remain distributed across the channel.

**Figure 10:**
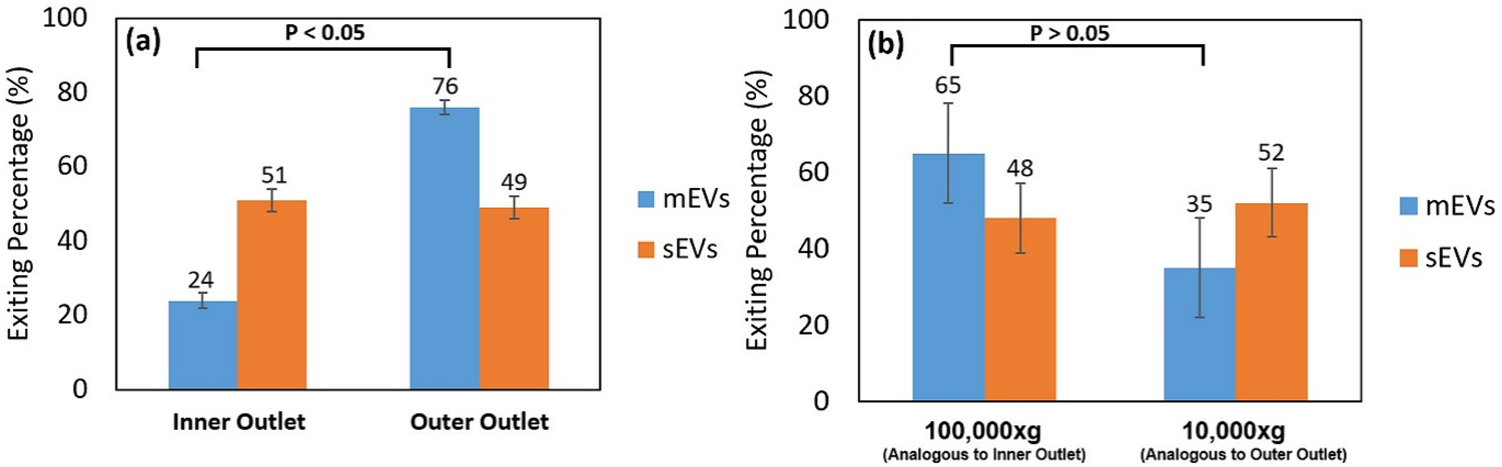
Results of **(a)** elasto-inertial focusing test on the mixture of U87 sEVs and U87 mEVs (test B4). Exiting percentage of particles from the inner outlet (left) and the outer outlet (right). n = 3, error bars = standard deviation (SD). **(b)** Re-isolation of U87 mEVs and sEV from a mixture U87 mEVs and U87 sEVs. sEV and mEV retrieval at 110,000×*g* and 10,000×*g* is equivalent to "inner" and "outer" retrieval of sEVs and mEVs respectively using the spiral channel.

The results of test B4 evaluating sEVs, mEVs, and 0.03% PEO are shown in Fig. 10a. The findings show that most of the mEVs (˜76%) were enriched at the outer outlet of the channel since they focused toward the outer wall of the channel under the co-effect of viscoelastic focusing and Dean flow. In this case, the viscoelastic focusing force dominated the inertial focusing force and subsequently the mEVs were focused toward the centerline of the channel. The Dean flow drag force results in movement of the mEVs towards the channel outer sidewall since the flow direction is toward the channel outer sidewall in the channel center. Being smaller, the sEVs were not affected by viscoelastic focusing, and their movement is dominated by Dean flow. As a result, sEVs had dispersed distribution and appeared in both outlets. This confirms that no sEV focusing occurred for the sEVs using the experimental test conditions. No focusing of sEVs resulted in the limited separation of sEVs and mEVs. The limited separation of sEVs and mEVs could also be a result of the overlapping size distributions of the original EVs populations as shown in Fig. 3.

The results of re-isolating of mEVs and sEV from a mixture of U87 mEVs and U87 sEV using elasto-inertial focusing versus differential ultracentrifugation are shown in Fig. 10. Comparing elasto-inertial focusing (Fig. 10a) and differential ultracentrifugation (Fig. 10b) re-isolation results demonstrate that not only did the spiral channel perform mEV and sEV re-isolation similar to differential ultracentrifugation on a miniaturized device scale, but it also resulted in significantly improved mEV re-isolation (considering the p values).

In differential ultracentrifugation, particles are isolated based on the differences in their densities. Theoretically, mEVs and sEVs were expected to be enriched at 10,000×*g* and 110,000×*g*, respectively. This is a result of mEVs having higher density than sEVs and thus requiring less centrifugal force for isolation. However, the experimental results (Fig. 10b) show the opposite. The mixing of mEV and sEV populations following re-isolation using differential ultracentrifugation can best be explained by a combination of low innate EV zeta (ζ) potential, and EV damage and aggregation induced by the differential ultracentrifugation process. Previously we determined that U87 mEVs and sEVs have very low ζ potentials (between 0 and –15 mV) in physiologic buffers such as PBS^51^. The ζ potential of an EV is determined at the slipping plane or electrical double layer interface of ions separating the vesicle surface from the fluid (PBS) in contact with the vesicle surface. For traditional nanoparticle formulations, to minimize particle aggregation or flocculation and maximize colloidal stability, ζ potentials ≤ −30 mV or ≥  + 30 mV are desired^49^. The closer an EV ζ potential is to 0 mV, the more readily an EV population aggregates. U87 mEVs and sEVs with ζ potentials between 0 and −15 mV have high innate aggregation potential.

Further, it is well known that one limitation of isolating EVs by means of differential ultracentrifugation is that the process induces EV damage and aggregation^40,41,60^. This can easily be observed on TEM^60^. TEM of U87 EVs (Fig. 4) demonstrates some broken EV “C-shaped” fragments and aggregates, particularly for sEVs (Fig. 4c). Disruption of the vesicle surface would be expected to change the particle ζ potential since the voltage potential across the EV membrane bilayer would be decreased. As a result, damaged EVs would further facilitate EV aggregation during differential ultracentrifugation.

In such a scenario, aggregates of damaged sEVs isolated by the first round of differential ultracentrifugation are more susceptible to pelleting at 10,000×*g* since the larger aggregates are less soluble, readily precipitate and pellet with the mEVs at 10,000×*g* (Fig. 10b, 10,000×*g*). In contrast, damage to mEVs at 10,000×*g*, resulting in low recovery (Fig. 6a), artificially generates some smaller broken mEVs with lost internal contents having less density, and/or even less stable ζ potentials. These low density damaged mEVs aggregate with similarly less dense and/or soluble sEVs that are not susceptible to isolation at 10,000×*g* but are susceptible to isolation at 110,000×*g* resulting in co-isolation of the mEVs with the sEVs at 110,000×*g* (Fig. 10b, 110,000×*g*).

**Figure 11:**
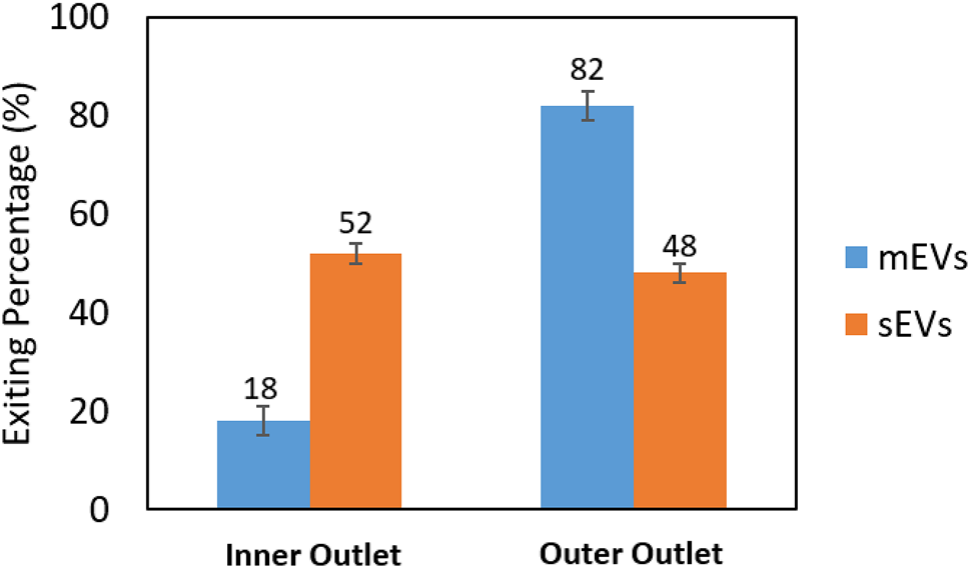
Results of inertial focusing test on the mixture of U87 sEVs and U87 mEVs (test B5). **(a)** Exiting percentage of particles from the inner outlet (left) and the outer outlet (right) **(b)**. n = 3, error bars = standard deviation (SD).

**Figure 12:**
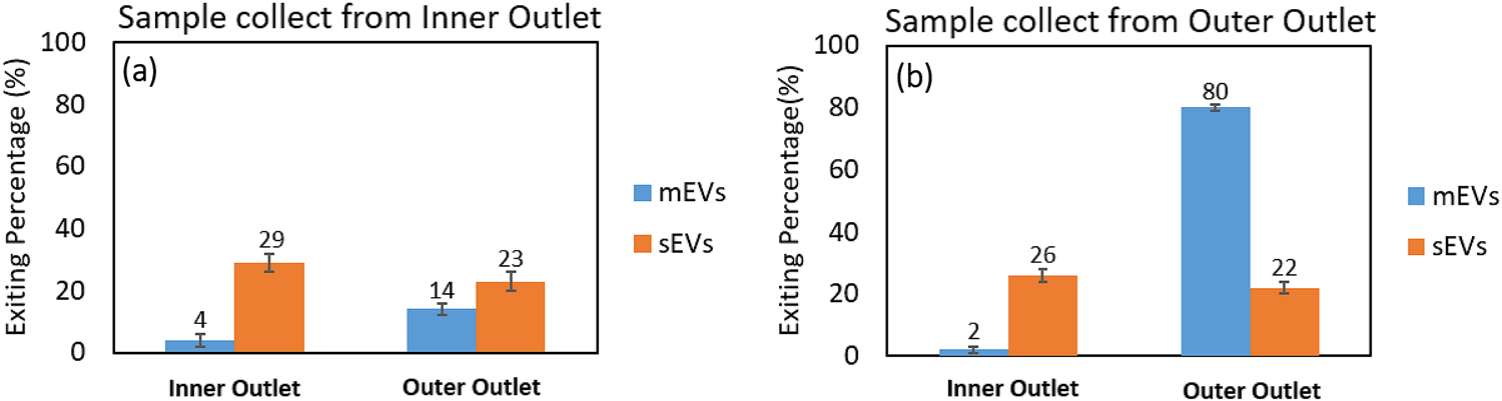
The results of recycling test done for the test B5R for the first-pass sample collected from the inner outlet **(a)** and the first-pass sample collected from the outer outlet **(b)**. n = 3, error bars = standard deviation (SD).

In contrast, elasto-inertial focusing of EVs results in less damage and aggregation of EVs than differential ultracentrifugation. This results in more colloidal stability of the mEV and sEV mixture, particularly mEVs. The mEVs do not as readily aggregate with sEVs beyond their innate aggregation potential. In our previous study, while not significantly different, U87 mEVs did show a modest trend in more stable ζ potential than U87 sEVs^51^, that further supports the afore mentioned scenario. The results of test B5, focusing sEVs and mEVs with 0.03% PEO are shown in Fig. 11, respectively. Comparing the results of test B4 and B5 indicate that by supplying a higher flow rate, a greater percentage of large particles (mEVs) were focused toward the outer outlet of the channel. As a result, the separation efficiency was improved. The result of the recycling test (Test B5R) is shown in Fig. 12. In this test, the large particles (mEVs) collected from the outer outlet during the first pass through are almost fully focused to the outer half of the channel (outer outlet) during the second pass through since particles size is further skewed toward the larger distribution after the first particle collection. Considering the percentages of particles obtained from the outer outlet for the recycling test (sample collected from the outer outlet, Fig. 12b), post one round of recycling, 80% of mEVs were separated with 22% contamination of sEVs. Alternatively, based on the sum of the percentages of particles recovered from the inner outlets, post one round of recycling (Fig. 12a,b), 55% of sEVs and 6% of mEVs were recovered.

## Conclusion

In this work, the capability of elasto-inertial focusing was studied for the separation of sEVs from mEVs. First, the focusing of 1000 nm polystyrene particles was investigated. The results indicated that for 1000 nm particles, fully focused streams were observed. The FWHM of the focusing was decreased from 7.1 to 5.9 µm, as the concentration of PEO increased from 0.01 to 0.03% and most of the background signal was eliminated resulting in more particle focusing. The optimized microbead separation parameters were applied for the purification of sEVs from mEVs. Good mEV focusing was achieved. However, the focusing is less than that of the polystyrene particles as a result of the polydisperse nature of the mEVs. Still, 55% of sEVs were recovered with 6% contamination of mEVs and 80% of mEVs were recovered with 22% contamination of sEVs after doing one-round of recycling. In contrast, recovery of U87 sEVs and mEVs reisolated using a typical second centrifugation wash step was only 53% and 8%, respectively (Fig. 6). Based on the flow rate during the separation process, it was possible to process 5 ml of sample in 55 min. This processing time can be further reduced by applying several devices in parallel. The separation efficiency can be improved by improving the focusing of mEVs or by potentially focusing sEVs. However, a much smaller channel (with reduced throughput) would likely be required to focus sEVs. Small EVs might also be potentially focused by increasing the inlet flow rate or increasing the concentration of PEO. The use of a spiral channel to focus mEVs enabled the purification of both mEVs and sEVs from a mixture of the two EV types.

We did not achieve 100% re-separation of U87 mEVs and sEVs compared to the initial isolation by differential ultracentrifugation. However, our spiral channel separation process resulted in greater mEV and sEV recovery compared to a second round of mEV and sEV purification or washing using differential ultracentrifugation. Recovery of mEVs more so than sEVs, using the spiral channel, was much better than a second round of centrifugation (Fig. 6). The spiral channel performed similar to differential ultracentrifugation in reisolating sEVs while significantly improving mEV reisolation from a mixture of sEVs and mEVs (Fig. 10). It’s possible that increased recovery and separation of mEVs by the spiral channel is a result of elasto-inertial flow focusing applying a gentler separation force to the EVs than the high g forces applied during differential ultracentrifugation resulting in more EV aggregation, damage and loss.

The key benefit of our elasto-inertial focusing system is that it is a novel compact microfluidic device that mechanistically works like gold standard differential ultracentrifugation for EV isolation. Because of its compact size, it can potentially be integrated with other microfluidic lab-on-a-chip and similar technologies. Integrated microfluidic elasto-inertial focusing can enable scalable, continuous separation of small microliter to milliliter volume limited samples upstream or downstream on EV chip-based detection and capture devices. This is not possible using machines the size of an ultracentrifuge. The elasto-inertial focusing technology is also mechanically simple with few moving parts and low cost to produce. As a result, one potential application includes integration of microfluidic elasto-inertial focusing into small clinical bedside point of care devices to detect clinical laboratory EV-based biomarkers in patient biofluids. A second application of the technology is that it can also be integrated into closed, sterile microfluidic biofluid processing systems. Spiral channels can be positioned downstream of compact, low mass cell culture bioreactors to obtain EVs as they are produced under sterile conditions. This is particularly relevant to the study of pathogenic EVs and viruses and could be applicable to BSL3 and BSL4 facilities to enable closed lab-on-a-chip experiments using less virions to minimize expenditures and increase biosafety. We believe with further development; our spiral channel technology can also be coupled to other chip-based microfluidic EV isolation methods in series and/or parallel to enable retrieval of EVs from various biofluids based on label-free physical EV properties and label-dependent EV affinity capture technologies. Combined, these methods will fine-tune EV isolation and interrogation, and minimize loss of EV types and subtypes to advance EV-based biomarker and therapeutic development.

## Supplementary Information

Supplementary Information.

## Data Availability

All relevant data generated or analyzed for this study are available within the article and the associated Supplementary Information. Any other data are available from F.S. (farhad.shiri@utah.edu) upon reasonable request.

## References

[1] Corrado C (2013). Exosomes as intercellular signaling organelles involved in health and disease: Basic science and clinical applications. Int. J. Mol. Sci..

[2] Hood JL, Pan H, Lanza GM, Wickline SA (2009). Paracrine induction of endothelium by tumor exosomes. Lab. Invest..

[3] Hood JL (2019). Natural melanoma-derived extracellular vesicles. Semin. Cancer Biol..

[4] Baranyai T (2015). Isolation of exosomes from blood plasma: Qualitative and quantitative comparison of ultracentrifugation and size exclusion chromatography methods. PLoS ONE.

[5] Hood JL, San RS, Wickline SA (2011). Exosomes released by melanoma cells prepare sentinel lymph nodes for tumor metastasis. Cancer Res..

[6] Minciacchi VR (2015). Large oncosomes contain distinct protein cargo and represent a separate functional class of tumor-derived extracellular vesicles. Oncotarget.

[7] Di Vizio D (2012). Large oncosomes in human prostate cancer tissues and in the circulation of mice with metastatic disease. Am. J. Pathol.

[8] Grant R (2011). A filtration-based protocol to isolate human plasma membrane-derived vesicles and exosomes from blood plasma. J. Immunol. Methods.

[9] Cheruvanky A (2007). Rapid isolation of urinary exosomal biomarkers using a nanomembrane ultrafiltration concentrator. Am. J. Physiol. Renal Physiol..

[10] Petersen KE (2014). A review of exosome separation techniques and characterization of B16–F10 mouse melanoma exosomes with AF4-UV-MALS-DLS-TEM. Anal. Bioanal. Chem..

[11] Zhang H, Lyden D (2019). Asymmetric-flow field-flow fractionation technology for exomere and small extracellular vesicle separation and characterization. Nat. Protoc..

[12] Ravi RK, Khosroheidari M, DiStefano JK (2015). A modified precipitation method to isolate urinary exosomes. J. Vis. Exp..

[13] Zeringer E, Barta T, Li M, Vlassov AV (2015). Strategies for isolation of exosomes. Cold Spring Harbor Protoc..

[14] Wang Z (2013). Ciliated micropillars for the microfluidic-based isolation of nanoscale lipid vesicles. Lab Chip.

[15] Lee K, Shao H, Weissleder R, Lee H (2015). Acoustic purification of extracellular microvesicles. ACS Nano.

[16] Liu C (2019). λ-DNA-and aptamer-mediated sorting and analysis of extracellular vesicles. J. Am. Chem. Soc..

[17] Zhou Y, Ma Z, Tayebi M, Ai Y (2019). Submicron particle focusing and exosome sorting by wavy microchannel structures within viscoelastic fluids. Anal. Chem..

[18] Bhagat AAS, Kuntaegowdanahalli SS, Papautsky I (2008). Continuous particle separation in spiral microchannels using dean flows and differential migration. Lab Chip.

[19] Kuntaegowdanahalli SS, Bhagat AAS, Kumar G, Papautsky I (2009). Inertial microfluidics for continuous particle separation in spiral microchannels. Lab Chip.

[20] Yoon DH (2009). Size-selective separation of micro beads by utilizing secondary flow in a curved rectangular microchannel. Lab Chip.

[21] Sun J (2012). Double spiral microchannel for label-free tumor cell separation and enrichment. Lab Chip.

[22] Sun J (2013). Size-based hydrodynamic rare tumor cell separation in curved microfluidic channels. Biomicrofluidics.

[23] Warkiani ME (2014). Slanted spiral microfluidics for the ultra-fast, label-free isolation of circulating tumor cells. Lab Chip.

[24] Wu L, Guan G, Hou HW, Bhagat AAS, Han J (2012). Separation of leukocytes from blood using spiral channel with trapezoid cross-section. Anal. Chem..

[25] Di Carlo D, Irimia D, Tompkins RG, Toner M (2007). Continuous inertial focusing, ordering, and separation of particles in microchannels. PNAS.

[26] Di Carlo D, Edd JF, Irimia D, Tompkins RG, Toner M (2008). Equilibrium separation and filtration of particles using differential inertial focusing. Anal. Chem..

[27] Goda K (2012). High-throughput single-microparticle imaging flow analyzer. PNAS.

[28] Zhang J (2014). Inertial particle separation by differential equilibrium positions in a symmetrical serpentine micro-channel. Sci. Rep..

[29] Zhang J, Yan S, Li W, Alici G, Nguyen N-T (2014). High throughput extraction of plasma using a secondary flow-aided inertial microfluidic device. RSC Adv..

[30] Zhang J, Li W, Li M, Alici G, Nguyen N-T (2014). Particle inertial focusing and its mechanism in a serpentine microchannel. Microfluid. Nanofluid..

[31] Amini H (2013). Engineering fluid flow using sequenced microstructures. Nat. Commun..

[32] Nunes JK (2014). Fabricating shaped microfibers with inertial microfluidics. Adv. Mater..

[33] Zhou J, Papautsky I (2013). Fundamentals of inertial focusing in microchannels. Lab Chip.

[34] Bhagat AAS, Kuntaegowdanahalli SS, Papautsky I (2008). Enhanced particle filtration in straight microchannels using shear-modulated inertial migration. Phys. Fluids.

[35] Ciftlik AT, Ettori M, Gijs MA (2013). High throughput-per-footprint inertial focusing. Small.

[36] Son J (2015). Non-motile sperm cell separation using a spiral channel. Anal. Methods..

[37] Son J, Samuel R, Gale BK, Carrell DT, Hotaling JM (2017). Separation of sperm cells from samples containing high concentrations of white blood cells using a spiral channel. Biomicrofluidics.

[38] Tay HM (2017). Rapid purification of sub-micrometer particles for enhanced drug release and microvesicles isolation. NPG Asia Mater..

[39] Greening, D. W., Xu, R., Ji, H., Tauro, B. J. & Simpson, R. J. *Proteomic Profiling*. 179–209. (Springer, 2015).

[40] Livshits MA (2015). Isolation of exosomes by differential centrifugation: Theoretical analysis of a commonly used protocol. Sci. Rep..

[41] Brennan K (2020). A comparison of methods for the isolation and separation of extracellular vesicles from protein and lipid particles in human serum. Sci. Rep..

[42] Amini H, Lee W, Di Carlo D (2014). Inertial microfluidic physics. Lab Chip.

[43] Xiang N, Ni Z, Yi H (2018). Concentration-controlled particle focusing in spiral elasto-inertial microfluidic devices. Electrophoresis.

[44] Leshansky AM, Bransky A, Korin N, Dinnar U (2007). Tunable nonlinear viscoelastic “focusing” in a microfluidic device. Phys. Rev. Lett..

[45] Feng H (2022). Viscoelastic particle focusing and separation in a spiral channel. Micromachines.

[46] Zhou, Y., Ma, Z. & Ai, Y. Dynamically tunable elasto-inertial particle focusing and sorting in microfluidics. *Lab Chip* (2019). 10.1039/c9lc01071h 31894813

[47] Feng H, Magda JJ, Gale BK (2019). Viscoelastic second normal stress difference dominated multiple-stream particle focusing in microfluidic channels. Appl. Phys. Lett..

[48] Li D, Lu X, Xuan X (2016). Viscoelastic separation of particles by size in straight rectangular microchannels: A parametric study for a refined understanding. Anal. Chem..

[49] Hood JL, Scott MJ, Wickline SA (2014). Maximizing exosome colloidal stability following electroporation. Anal. Biochem..

[50] Bardi GT, Al-Rayan N, Richie JL, Yaddanapudi K, Hood JL (2019). Detection of inflammation-related melanoma small extracellular vesicle (sEV) mRNA content using primary melanocyte sEVs as a reference. Int. J. Mol. Sci..

[51] Shiri F (2020). Characterization of human glioblastoma versus normal plasma-derived extracellular vesicles preisolated by differential centrifugation using cyclical electrical field-flow fractionation. Anal. Chem..

[52] Cirelli, R. A., Watson, G. P. & Nalamasu, O. *Encyclopedia of Materials: Science and Technology* (eds. Jürgen Buschow, K. H. *et al.*) 6441–6448 (Elsevier, 2001).

[53] Franssila, S. & Tuomikoski, S. *Handbook of Silicon Based MEMS Materials and Technologies* (eds. Lindroos, V., Tilli, M., Lehto, A. & Motooka, T.). 333–348. (William Andrew Publishing, 2010).

[54] Morello M (2013). Large oncosomes mediate intercellular transfer of functional microRNA. Cell Cycle.

[55] Hood JL (2018). Pre-analytical influences on the population heterogeneity of human extracellular vesicles sourced for nanomedicine uses. Nanomedicine (Lond).

[56] D'Asti E (2012). Oncogenic extracellular vesicles in brain tumor progression. Front. Physiol..

[57] Bandu R, Oh JW, Kim KP (2019). Mass spectrometry-based proteome profiling of extracellular vesicles and their roles in cancer biology. Exp. Mol. Med..

[58] Kowal J (2016). Proteomic comparison defines novel markers to characterize heterogeneous populations of extracellular vesicle subtypes. Proc. Natl. Acad. Sci. U. S. A..

[59] Jeppesen DK (2019). Reassessment of exosome composition. Cell.

[60] Linares R, Tan S, Gounou C, Arraud N, Brisson AR (2015). High-speed centrifugation induces aggregation of extracellular vesicles. J. Extracell. Vesicles.

