# Supplementary material for: Separation of U87 glioblastoma cell-derived small and medium extracellular vesicles using elasto-inertial flow focusing (a spiral channel)
Source: Sci Rep. 2022 Apr 12;12:6146. doi: 10.1038/s41598-022-10129-8 (PMC9005724; doi:10.1038/s41598-022-10129-8)
Supplement: Supplementary file 1 — Supplementary Information. [file 41598_2022_10129_MOESM1_ESM.docx]

**Supplementary Information**

**Separation of U87 glioblastoma cell-derived small and medium extracellular vesicles using elasto-inertial flow focusing (A spiral channel)**

Farhad Shiri^1†^, Haidong Feng^1^, Kevin E. Petersen^1^, Himanshu Sant^1^, Gina T. Bardi^2^, Luke A. Schroeder^2^, Michael L. Merchant^5^, Bruce K. Gale^1†^, Joshua L. Hood^2,3,4*^

^1^ University of Utah, Department of Mechanical Engineering, Salt Lake City, UT 84112

^2^ University of Louisville, School of Medicine, Department of Pharmacology and Toxicology, Louisville, KY 40202

^3^ University of Louisville, James Graham Brown Cancer Center, Louisville, KY 40202

^4^ University of Louisville, Hepatobiology and Toxicology COBRE, Louisville, KY 40202

^5^ University of Louisville, Department of Medicine, Division of Nephrology & Hypertension, Louisville, KY 40202

*^†^Corresponding Authors: Joshua L. Hood (Email: [joshua.hood@louisville.edu](mailto:joshua.hood@louisville.edu)), Bruce K. Gale (Email: [bruce.gale@utah.edu](mailto:bruce.gale@utah.edu))

**Methods**

**Extracellular vesicle culture and isolation.** Isolation of EVs from cell culture by means of differential ultracentrifugation has previously been established ^1-4^. EVs were isolated from U87 glioma cells (ATCC^®^ HTB-14^™^) at the University of Louisville. Cells were grown to 70% confluence in three 300 cm^2^ flasks. Culture media was removed, and the cells were washed with DMEM. U87 cells were then cultured for 48 h in bovine EV-free conditioned media. Conditioned media was prepared by subjecting normal culture media to overnight ultracentrifugation at 110,000 x g to remove bovine EVs ^5^. Post 48 h cellular growth in conditioned media, conditioned media was removed, diluted 1:1 in 1X PBS ^6^, and processed using differential ultracentrifugation with a Type 50.2 Ti rotor (Beckman Coulter Inc., Brea, CA, USA) ^5^. Supernatants were collected after each round of centrifugation, and the pellets were discarded. Centrifugation at 3400 x g for 30 min was used to remove residual cells, debris ^7,8^ and larger-sized EVs ^2^. Subsequently, ultracentrifugation at 10,000 x g for 30 min was used to isolate mEVs ^2^. Immediately prior to the mEV isolation step, fluorescent lipophilic cationic tracer dyes were used to label EVs. Fluorescent lipophilic tracers DiI (1,10-dioctadecyl-3,3,30,30-tetramethylindocarbocyanine Ex. 549/Em. 565) and DiO (3,30-dihexadecyloxacarbocyanine Ex. 484/Em. 501) were used to dye U87 sEVs and mEVs respectively at a concentration of 1.0 µM according to established methods ^9,10^. Fluorescent dyes were not mixed for labeling, but rather different EV batches were labeled fluorescent red or green. So, if the EV population of interest was mEVs, then all mEVs and sEVs in an EV batch sample were dyed with DiO (fluorescent green). If the EV population of interest was sEVs, then all mEVs and sEVs in an EV batch sample were dyed with DiI (fluorescent red). Finally, the sEV pellet was collected after ultracentrifugation of the post mEV isolation supernatant at 110,000 x g for 1.5 h ^11^. EVs were stored in PBS at -80°C ^6^. Samples were shipped overnight and stored at -80°C until use. Polyethylene oxide (PEO) dissolved in 1 X PBS was used as the viscoelastic reagent in the spiral channel separation tests.

**Liquid chromatography–mass spectrometry (LC–MS) of EV protein content.** U87 cells were pelleted at 500xg and 4oC for 5min then lysed in 1mL 5% (w/v) sodium dodecylsulfate (SDS) in 0.05M triethylammonium bicarbonate (TEABC) pH 8.5 supplemented with 1X HALTTM protease/phosphatase inhibitor cocktail (Thermo) and 5mM EDTA and triturated 10 times using a 21g needle. Debris were pelleted by centrifugation at 13,000xg at 22oC for 8min. The supernatant was transferred to a clean microtube, and the protein concentration was estimated using a DC (detergent compatible) Assay (BioRad, Hercules, CA) against a bovine serum albumin standard. Aliquots (25 µg protein) of U87 mEV and sEV samples were adjusted to 5% SDS in 0.05M TEABC, pH 8.5 supplemented with 1X HALTTM protease/phosphatase inhibitor cocktail (Thermo Fisher Scientific, Waltham, MA) supplemented with 5mM EDTA. U87 cell lysate, mEV and sEV aliquots (25µg) were digested according to the S-trap Mini (Protifi, Farmingdale, NY, USA) protocol using mass spectrometry grade trypsin (Promega, Inc, Madison, WI) to sample ratio of 1:25. Digests were diluted to a final concentration of 0.1µg/µL in 2% v/v acetonitrile / 0.1% v/v formic acid.

**LC-MS data acquisition.** Sample aliquots (0.1 µg) were analyzed using an EASY n-LC (Thermo Fisher Scientific, Waltham, MA) UHPLC -LTQ-Velos-Orbitrap ELITE (Thermo Fisher Scientific, Waltham, MA) mass spectrometry system. The peptides were loaded onto a Dionex Acclaim PepMap 100 75µm x 2cm, nanoViper (C18, 3µm, 100Å) trap and separated with a 50 min 2% to 48% acetonitrile gradient on an Acclaim PepMap RSLC 50µm x 15cm, nanoViper (C18, 2µm, 100Å) separating column (Thermo Fisher Scientific, Waltham, MA) prior to the introduction by nanoelectrospray using a 40mm stainless steel emitter into a Nanospray Flex source (Thermo Fisher Scientific, Waltham, MA) into a LTQ-Velos-Orbitrap ELITE (Thermo Fisher Scientific, Waltham, MA) mass spectrometer with the ion transfer capillary temperature of 225°C, and the spray voltage of 1.75kV.

An LTQ-Velos-Orbitrap ELITE – ETD mass spectrometer (Thermo Fisher Scientific, Waltham, MA) was used to collect data from the LC eluate. An Nth Order Double Play was created in Xcalibur v2.2 (Thermo Fisher Scientific, Waltham, MA). Scan event one of the methods obtained an FTMS MS1 scan (normal mass range; 120,000 resolution, full scan type, positive polarity, profile data type) for the range 300-2000m/z. Scan event two obtained ITMS MS2 scans (normal mass range, rapid scan rate, centroid data type) on up to twenty peaks that had a minimum signal threshold of 5,000 counts from scan event one. A parent list using m/z values as masses was used to trigger the acquisition on ions within ±15ppm of monoisotopic masses from the UniprotKB accessions P62330, O43707, P21926, P08962, P60033, Q99816, O00560, and P08758. For each accession, the three peptides with the highest ESS scores were selected from distinctly observed peptides in the peptideatlas.org. Masses were calculated in Protein Calculator in Xcalibur 2.2 assuming Carbamidomethyl(C) as static and Oxidation(M) as variable for these peptides. If no parent masses were found, the most intense ions were fragmented. The lock mass option was enabled (0% lock mass abundance) using the 371.101236m/z polysiloxane peak as an internal calibrant.

**LC-MS data analysis.** Proteome Discoverer v2.4.0.305 (Thermo Fisher Scientific, Waltham, MA) was used to analyze the data collected by the mass spectrometer. The 10/01/2021 version of the UniprotKB reviewed reference proteome canonical and isoform Homo sapiens (taxonomic identifier UP000005640) sequences were used in the Mascot v2.5.1 and SequestHT searches. Specified criteria for the search included: trypsin cleavage (maximum two missed cleavages; inhibition by P), static modification of cysteine by carbamidomethyl, and variable oxidation of methionine, acetylation (Acetyl) of the protein N-term, N-terminal methionine (Met-) loss, and Met-loss+Acetyl (Protein N-term M) as dynamic. MS2 fragment mass accuracy was set to 0.6Da (monoisotopic) and MS1 parent tolerance was set to 10ppm (monoisotopic). A Percolator PSM Validator node was included in the workflow to support false discovery rate estimations.

The result files from Proteome Discoverer were loaded into Scaffold Q+S v5.0.1 (Proteome Software Inc., Portland, OR, USA) using Precursor Intensity (Standard) and the prefiltered Scoring System mode using the Percolator FDR results. FDR thresholds were pre-computed and protein accession numbers were annotated with the Scaffold default NCBI GO terms. Proteins were grouped into clusters to satisfy the parsimony principle. Proteins and peptides were filtered at 1% FDR in Scaffold with a minimum number of one peptide per protein.

**Optimization of focusing of 1000 nm PS microbeads.** A series of tests were done to optimize the flow conditions and the viscoelastic parameters (PEO concentration) for the focusing of 1000 nm microbeads in the spiral channel. For this series of experiments, the focusing of the microbeads was tested under different flow rates and PEO concentrations. The total inlet flow rate of the sample was altered from 0.04 ml/min to 0.08 ml/min with 0.01% PEO and then the PEO concentration was increased to 0.03%. The influence of inertial flow and viscoelastic flow is characterized by Re and Wi as described in the theory session of the main text. For all tests, the focusing of microbeads was observed and recorded using a fluorescent microscope. The parameters and the focusing results of the optimization tests are reported in Table S1.

**Table S1.** Conditions for optimization of the focusing tests of PS particles in the spiral channel

| **Test Number** | **Particle Diameter (nm)** | **Total Channel Flow Rate (mL/min)** | **PEO concentration** | **Re** | **Wi** | **Channel exit splitting ratio (Inner : Outer)** | **Percentage of particles focused to the outer half of the channel** | **Results shown in Figure** |
| --- | --- | --- | --- | --- | --- | --- | --- | --- |
| S1 | 1000 | 0.04 | 0.01% | 9.392 | 57 | 50:50 | 52% | S1 |
| S2 | 1000 | 0.05 | 0.01% | 11.740 | 71.25 | 50:50 | 58% | S2 |
| S3 | 1000 | 0.06 | 0.01% | 14.088 | 85.5 | 50:50 | 67% | S3 |
| S4 | 1000 | 0.07 | 0.01% | 16.437 | 99.75 | 50:50 | 79% | S4 |
| S5 | 1000 | 0.08 | 0.01% | 18.785 | 114 | 50:50 | 82% | S5 |
| S6 | 1000 | 0.08 | 0.03% | 15.828 | 227.2 | 50:50 | 96% | S6 |

**Characterization of the Fluorescence Detectors.** The detection and quantification of the EVs were performed using two fluorescence detectors. For DiI dyed sEVs and DiO dyed mEVs, the excitation/emission wavelengths were set at 549 nm/570 nm and 475 nm/ 505 nm, respectively.

A series of dilution tests were carried out to construct the calibration curve of the fluorescence detectors. To do that, the received sEVs and mEVs were diluted 10 times, 20 times, 50 times, 100 times, 200 times, and 1000 times with 1xPBS buffer. The concentration of the received sEVs and mEVs were 0.73 µg/ml and 0.12 µg/ml, respectively. For each dilution factor, the fluorescence intensity was measured using the fluorescence detectors that were set for the detection of sEVs and mEVs and then the calibration curve (fluorescence detector signal versus the concentration of EVs) was plotted.

In addition to the calibration curve generation, a series of experiments were performed to determine if the fluorescence detectors were able to distinguish the sEVs from mEVs. To do this determination, sEVs and mEVs were injected into the fluorescence detectors separately and as a mixture. For the mixture of sEVs and mEVs, the concentrations of sEVs and mEVs were the same as when they were injected separately into the detectors.

**Results and discussion**

The results of the optimization of the flow conditions and viscoelastic parameters for the focusing of 1000 nm microbeads are shown in Figures S1 to S6.

According to the results, as the total inlet flow rate increased, more particles were focused toward the outer half of the channel at the outlet. The analysis of the light intensity of the recorded fluorescence images indicates that 52%, 58%, 67%, 79%, and 82% of 1000 nm microbeads were focused to the outer half of the channel under the total inlet flow rates of 0.04, 0.05, 0.06, 0.07, and 0.08 ml/min and in 0.01% PEO solution, respectively. The focusing was improved when the concentration of PEO was increased from 0.01% to 0.03% at the total flow rate of 0.08 ml/min. For 0.03% PEO, 96% of 1000 nm microbeads were focused within the outer half of the channel.


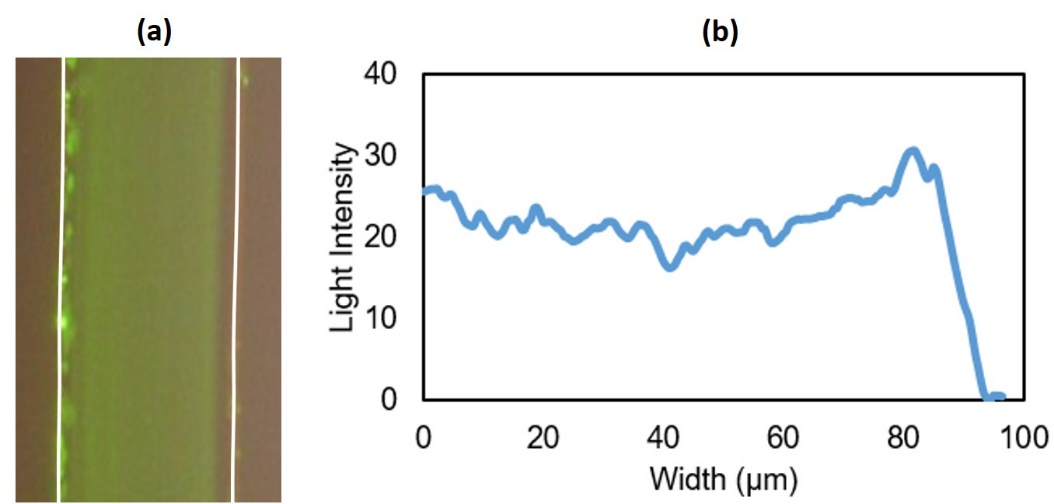


**Figure S1**. Results of 1000 nm polystyrene particle location imaging test (test S1) at the outlet under the total inlet flow rate of 0.04 ml/min and 0.01% PEO a) Image taken by a fluorescent microscope (the walls of the channel are marked by the white lines), b) the light intensity across the width of the channel based on the image in (a). 52% of microbeads were found within the outer half of the channel at the outlet of the channel.


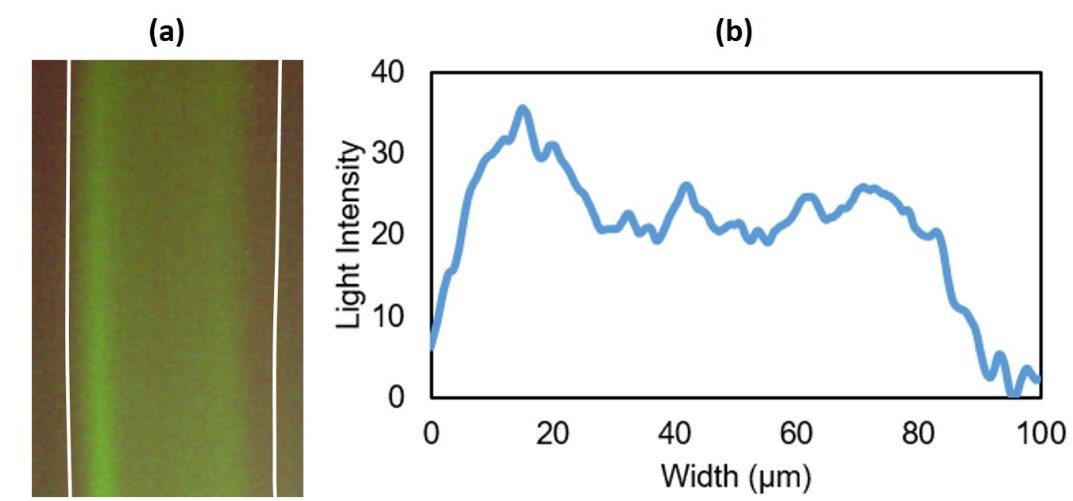


**Figure S2**. Results of 1000 nm polystyrene particle location imaging test (test S2) at the outlet under the total inlet flow rate of 0.05 ml/min and 0.01% PEO a) Image taken by a fluorescent microscope (the walls of the channel are marked by the white lines), b) the light intensity along the width of the channel based on the image in (a). 58% of microbeads were focused within the outer half of the channel at the outlet of the channel.


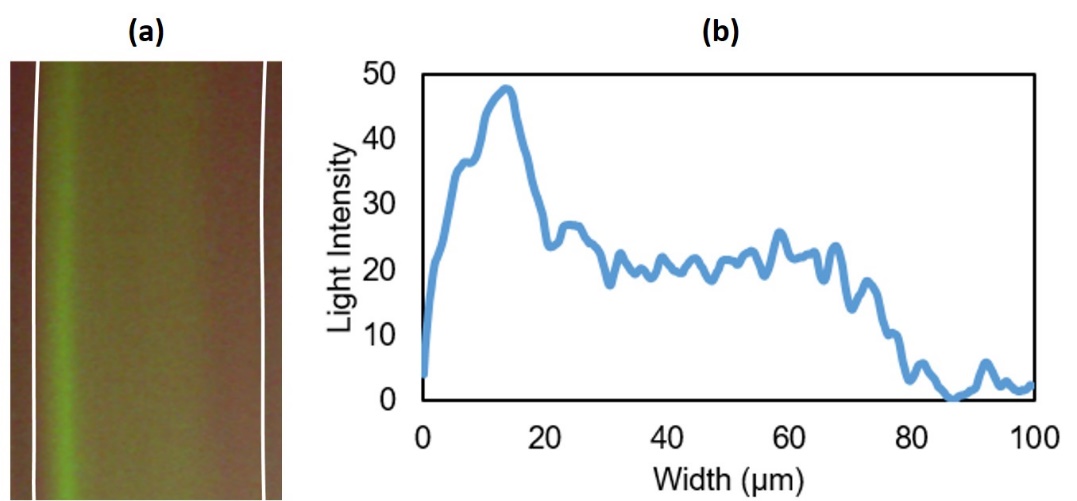


**Figure S3**. Results of 1000 nm polystyrene particle location imaging test (test S3) at the outlet under the total inlet flow rate of 0.06 ml/min and 0.01% PEO a) Image taken by a fluorescent microscope (the walls of the channel are marked by the white lines), b) the light intensity along the width of the channel based on the image in (a). 67% of microbeads were focused within the outer half of the channel at the outlet of the channel.


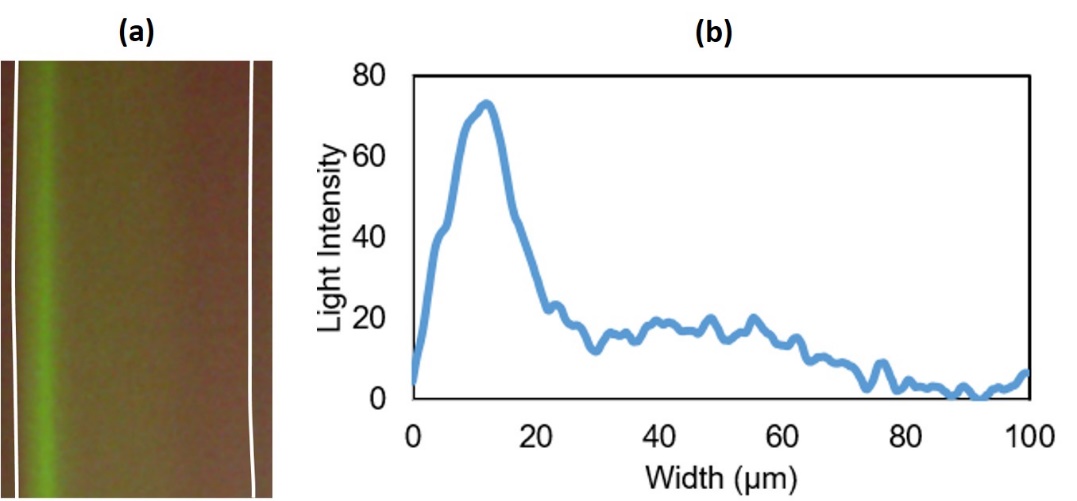


**Figure S4**. Results of 1000 nm polystyrene particle location imaging test (test S4) at the outlet under the total inlet flow rate of 0.07 ml/min and 0.01% PEO a) Image taken by a fluorescent microscope (the walls of the channel are marked by the white lines), b) the light intensity along the width of the channel based on the image in (a). 79% of microbeads were focused within the outer half of the channel at the outlet of the channel.


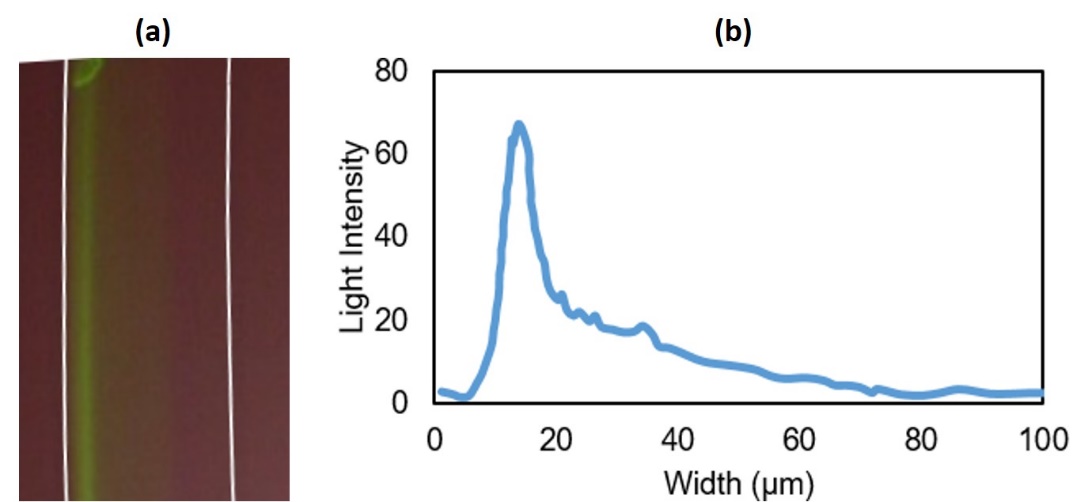


**Figure S5.** Results of 1000 nm polystyrene particle location imaging test (test S5) at the outlet under the total inlet flow rate of 0.08 ml/min and 0.01% PEO a) Image taken by a fluorescent microscope (the walls of the channel are marked by the white lines), b) the light intensity along the width of the channel based on the image in (a). 82% of microbeads were focused within the outer half of the channel at the outlet of the channel.


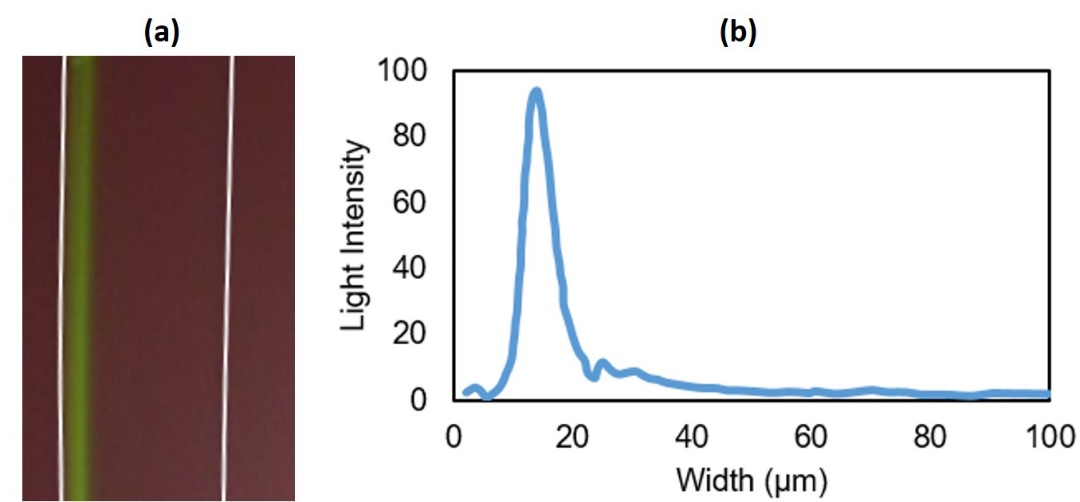


**Figure S6.** Results of 1000 nm polystyrene particle location imaging test (test S6) at the outlet under the total inlet flow rate of 0.08 ml/min and 0.03% PEO a) Image taken by a fluorescent microscope (the walls of the channel are marked by the white lines), b) the light intensity along the width of the channel based on the image in (a). 96% of microbeads were focused within the outer half of the channel at the outlet of the channel.

The results of the calibration curves of DiI dyed sEVs and DiO dyed mEVs are shown in Figure S7 and Figure S8. A linear correlation was found between the concentrations of DiI dyed sEVs and DiO dyed mEVs and the signal of the fluorescence detectors over the range of concentrations used for the separation process of sEVs and mEVs. This result indicates that the fluorescence signal of the detector can be used for the quantification of EVs exiting from two outlet ports of the spiral channel.


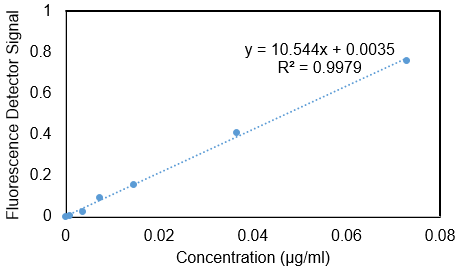


**Figure S7.** Calibration curve of DiI dyed sEVs


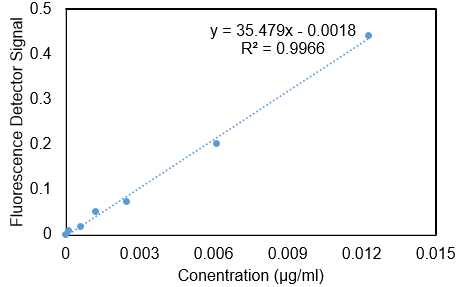


**Figure S8.** Calibration curve of DiO dyed mEVs

In Figure S9a and S9b, the signals of the fluorescence detectors that are set for the detection of DiI dyed sEVs and DiO dyed mEVs are shown, respectively. For these two figures, the sEVs and mEVs were injected separately (sEVs for Figure S9a and mEVs for Figure S9b). Figure S9c show the signals of the fluorescence detectors obtained from the injection of the mixture of sEVs and mEVs. Overall, the results show that the detectors were able to distinguish the sEVs from mEVs and that the fluorescence intensity obtained from individual injection of sEVs and mEVs (separate injection of sEVs and mEVs) corresponded to the detector signals when the sEVs and mEVs were injected together (mixture of sEVs and mEVs).


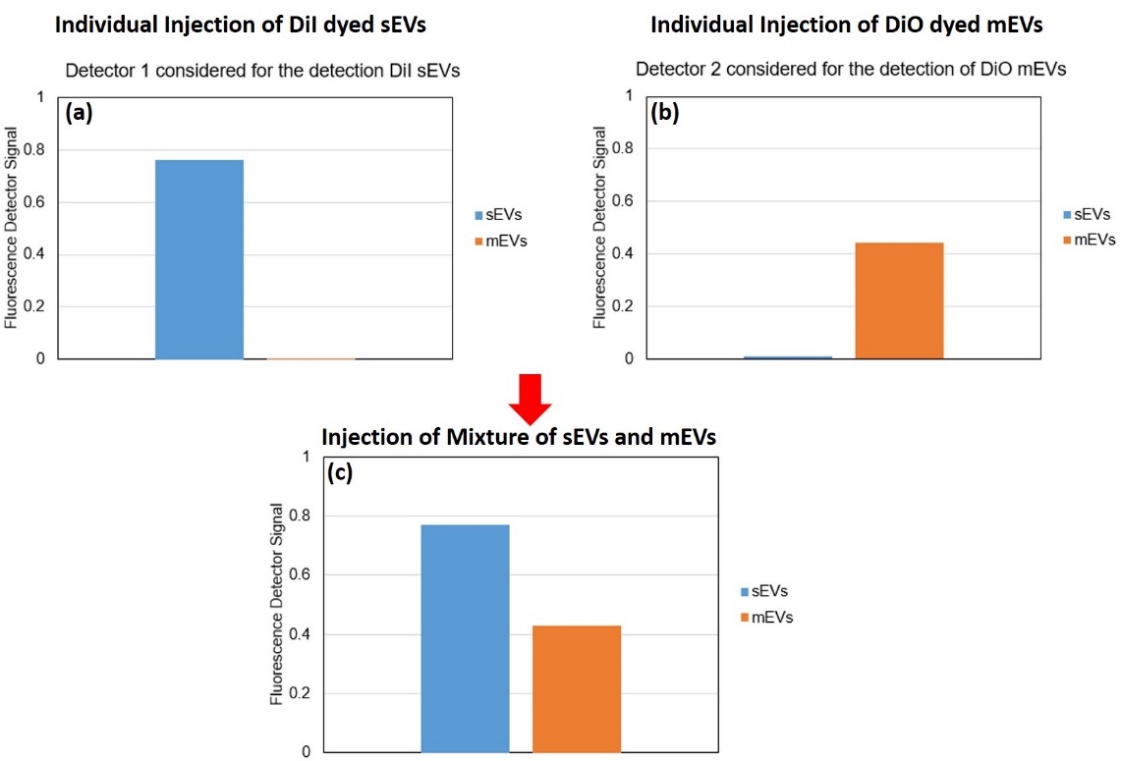


**Figure S9.** The fluorescence detector signals when a) DiI dyed sEVs were injected separately b) DiO dyed mEVs were injected separately c) sEVs and mEVs were injected together (as a mixture)

**References**

1. Hood, J. L., Pan, H., Lanza, G. M. & Wickline, S. A. Paracrine induction of endothelium by tumor exosomes. *Lab. Invest.* **89**, 1317-1328, doi:10.1038/labinvest.2009.94 (2009).

2. Kowal, J. *et al.* Proteomic comparison defines novel markers to characterize heterogeneous populations of extracellular vesicle subtypes. *Proc. Natl. Acad. Sci. U. S. A.* **113**, E968-977, doi:10.1073/pnas.1521230113 (2016).

3. Bardi, G. T., Al-Rayan, N., Richie, J. L., Yaddanapudi, K. & Hood, J. L. Detection of Inflammation-Related Melanoma Small Extracellular Vesicle (sEV) mRNA Content Using Primary Melanocyte sEVs as a Reference. *Int. J. Mol. Sci.* **20**, doi:10.3390/ijms20051235 (2019).

4. Shiri, F. *et al.* Characterization of Human Glioblastoma versus Normal Plasma-Derived Extracellular Vesicles Preisolated by Differential Centrifugation Using Cyclical Electrical Field-Flow Fractionation. *Anal. Chem.* **92**, 9866-9876, doi:10.1021/acs.analchem.0c01373 (2020).

5. Thery, C., Clayton, A., Amigorena, S. & Raposo, G. Isolation and Characterization of Exosomes from Cell Culture Supernatants and Biological Fluids. *Curr. Protoc. Cell Biol.*, 3.22.21-23.22.29 (2006).

6. Hood, J. L., Scott, M. J. & Wickline, S. A. Maximizing exosome colloidal stability following electroporation. *Anal. Biochem.* **448**, 41-49, doi:10.1016/j.ab.2013.12.001 (2014).

7. Gould, S. J. & Raposo, G. As we wait: coping with an imperfect nomenclature for extracellular vesicles. *J Extracell Vesicles* **2**, doi:10.3402/jev.v2i0.20389 (2013).

8. Heijnen, H. F., Schiel, A. E., Fijnheer, R., Geuze, H. J. & Sixma, J. J. Activated platelets release two types of membrane vesicles: microvesicles by surface shedding and exosomes derived from exocytosis of multivesicular bodies and alpha-granules. *Blood* **94**, 3791-3799 (1999).

9. Hood, J. L., Pan, H., Eby, C. S. & Wickline, S. A. Detection and Analysis of Nanoscale Tumor Membrane Biomarkers. *Am. J. Clin. Pathol.* **132**, 460-461 (2009).

10. Hood, J. L., San, R. S. & Wickline, S. A. Exosomes released by melanoma cells prepare sentinel lymph nodes for tumor metastasis. *Cancer Res.* **71**, 3792-3801, doi:10.1158/0008-5472.CAN-10-4455 (2011).

11. Bardi, G. T., Smith, M. A. & Hood, J. L. Melanoma exosomes promote mixed M1 and M2 macrophage polarization. *Cytokine* **105**, 63-72, doi:10.1016/j.cyto.2018.02.002 (2018).
